# Transcriptome analysis reveals the complexity of alternative splicing regulation in the fungus *Verticillium dahliae*

**DOI:** 10.1186/s12864-017-3507-y

**Published:** 2017-02-06

**Authors:** Lirong Jin, Guanglin Li, Dazhao Yu, Wei Huang, Chao Cheng, Shengjie Liao, Qijia Wu, Yi Zhang

**Affiliations:** 1 Key Laboratory of Integrated Pest Management on Crops in Central China, Institute of Plant Protection and Soil Science, Hubei Academy of Agricultural Sciences, Wuhan, Hubei 430064 China; 2 Center for Genome Analysis, ABLife Inc., Optics Valley International Biomedical Park, Building 9-4, East Lake High-Tech Development Zone, 388 Gaoxin 2nd Road, Wuhan, Hubei 430075 China; 3 Laboratory for Genome Regulation and Human Heath, ABLife Inc., Optics Valley International Biomedical Park, Building 9-4, East Lake High-Tech Development Zone, 388 Gaoxin 2nd Road, Wuhan, Hubei 430075 China; 4 College of Life Science, Shaanxi Normal University, Xi’an, Shaanxi 710119 China; 5 Seqhealth Technology Co., Ltd, Wuhan, Hubei 430075 China

**Keywords:** Alternative splicing, Fungus, *Verticillium dahliae*, Intron retention

## Abstract

**Background:**

Alternative splicing (AS) regulation is extensive and shapes the functional complexity of higher organisms. However, the contribution of alternative splicing to fungal biology is not well studied.

**Results:**

This study provides sequences of the transcriptomes of the plant wilt pathogen *Verticillium dahliae*, using two different strains and multiple methods for cDNA library preparations. We identified alternatively spliced mRNA isoforms in over a half of the multi-exonic fungal genes. Over one-thousand isoforms involve TopHat novel splice junction; multiple types of combinatory alternative splicing patterns were identified. We showed that one *Verticillium* gene could use four different 5′ splice sites and two different 3′ donor sites to produce up to five mature mRNAs, representing one of the most sophisticated alternative splicing model in eukaryotes other than animals. Hundreds of novel intron types involving a pair of new splice sites were identified in the *V. dahliae* genome. All the types of AS events were validated by using RT-PCR. Functional enrichment analysis showed that AS genes are involved in most known biological functions and enriched in ATP biosynthesis, sexual/asexual reproduction, morphogenesis, signal transduction etc., predicting that the AS regulation modulates mRNA isoform output and shapes the *V. dahliae* proteome plasticity of the pathogen in response to the environmental and developmental changes.

**Conclusions:**

These findings demonstrate the comprehensive alternative splicing mechanisms in a fungal plant pathogen, which argues the importance of this fungus in developing complicate genome regulation strategies in eukaryotes.

**Electronic supplementary material:**

The online version of this article (doi:10.1186/s12864-017-3507-y) contains supplementary material, which is available to authorized users.

## Background

Next-generation sequencing technology has provided pioneering opportunities for deciphering novel mechanisms of gene/genome regulation through sequencing and analysis of the cDNA libraries generated from the whole transcriptome of specific populations of RNA in experimental cells and tissues [1–5]. In addition to transcriptional regulation, alternative splicing (AS) of the primary transcripts of protein-coding genes represents one central post-transcriptional regulatory mechanism in shaping the transcriptome diversity and proteome complexity of higher eukaryotic genomes. High throughput transcriptome sequencing revealed that almost 94% of genes are alternatively spliced in humans [6]. In plants, alternative splicing was estimated to be 60% in *Arabidopsis*, 52% in soybean, 40% in cotton, 40% in maize, and 33% in rice intron-containing genes [7–12]. However, alternative splicing was not extensively studied because of the underestimated introns in fungi [13]. In recent years, highly developed high-throughput sequencing has revealed more introns than previously anticipated [14, 15]. In contrast to the predominant exon-skipping events in animals, plant and fungi AS events were reported to be predominantly involved in intron retention, while the other AS forms are rare [14–18].

AS controls almost all aspects of biological processes in mammalian cells and is involved in many human diseases [19–22], it is logical to expect it plays a role in biological functions of fungi. *Psc*RXLR1 was the first example of a non-effector transformed to a functional effector protein by alternative splicing in *Pseudoperonospora cubensis* [23]. *UmRrm75* is probably involved in cell growth, morphogenesis, and pathogenicity in *Ustilago maydis*, which was reported to be regulated by AS [24]. In addition, genes involved in virulence in fungal pathogens were reported to be regulated by alternative splicing [25, 26]. However, the influence from AS on the transcriptome output in the lower eukaryotic genomes is still limited.

*Verticillium* species are among the most devastating fungal pathogens that cause vascular wilt worldwide in a broad range of plant hosts including economically important crops such as cotton, soybean and tomato, but no effective chemical pesticides are available due in part to its soil-borne nature [27]. The recent release of the draft *V. dahliae* genome sequence [28] enables a genome-wide investigation of genes and molecular mechanisms underlying the pathogenicity of *V. dahliae*.

Here we used deep sequencing technology to profile the transcriptomes of two *V. dahliae* strains. Two different methods were applied to generate cDNA libraries for Illumina sequencing platform, showing the expression of over 95% of the annotated genes under the in vitro vegetative growth condition. Using computational algorithms developed in this study, we showed that about 50% of the intron-containing genes of *V. dahliae* were potentially under alternative splicing regulation. In addition to the large amount of intron retention AS events expected, we revealed over a thousand of AS events covering most non-intron-retention AS types. Combined with the functional clustering results of the AS genes, our results strongly suggest that this plant fungal pathogen has acquired sophisticated AS mechanisms to maximize its protein encoding potential and to control essential biological functions such as mycelium development, sporulation, signal response etc., and thus very likely AS contributes to the pathogenicity after the fungus encounters its hosts.

## Results

### Transcriptome landscape of *V. dahliae* and the likely enrichment of secondary structures in 5′ untranslated regions

To explore the AS in *V. dahliae*, two closely-related *V. dahliae* (V991w and V991b) isolates were subjected to next-generation sequencing. The cDNA was prepared from the polyadenylated RNA and subjected to the high-throughput sequencing using Illumina GAIIx platform. We obtained a total of 1.37 and 1.43 millions of cDNA reads from the two strains, respectively, which unambiguously mapped onto the sequenced regions of the annotated genes (10,535) [28]. Both strains showed a major distribution of the reads in the protein-coding regions (~70%), and the remaining reads matched the non-coding regions including 5′ UTR, 3′ UTR and intron (Additional file 1: Figure S1A and Additional file 2: Table S1A).

Among the 10,535 annotated *V. dahliae* genes, 9657 genes (91.7%) obtained expression evidence, with 9073 and 9335 genes being detected in V991w and V991b, respectively (Additional file 1: Figure S1B). The average sequence depth per base of all gene regions reached 12.67, and the sequence coverage reached 50% for about two third of genes. The quantitative expression level of each gene was represented by the number of Reads Per Kilo base of exonic region per Million (RPKM) mapped reads in all genes, showing that the majority of the *V. dahliae* genes was expressed relatively low-abundantly (Additional file 1: Figure S1C). We noticed the sequence depth at the 5′ and 3′ UTR regions were significantly higher than that of the CDS regions, which seems to be a global phenomenon (Additional file 2: Table S1C, Additional file 1: Figure S2A-C). The sequence depth in the intronic regions was higher than expected too. This result could be caused by the enriched local RNA secondary structure in non-coding regions, which were more susceptible to RNase III digestion and selectively enriched in the cDNA libraries [29–31].

**Fig. 1:**
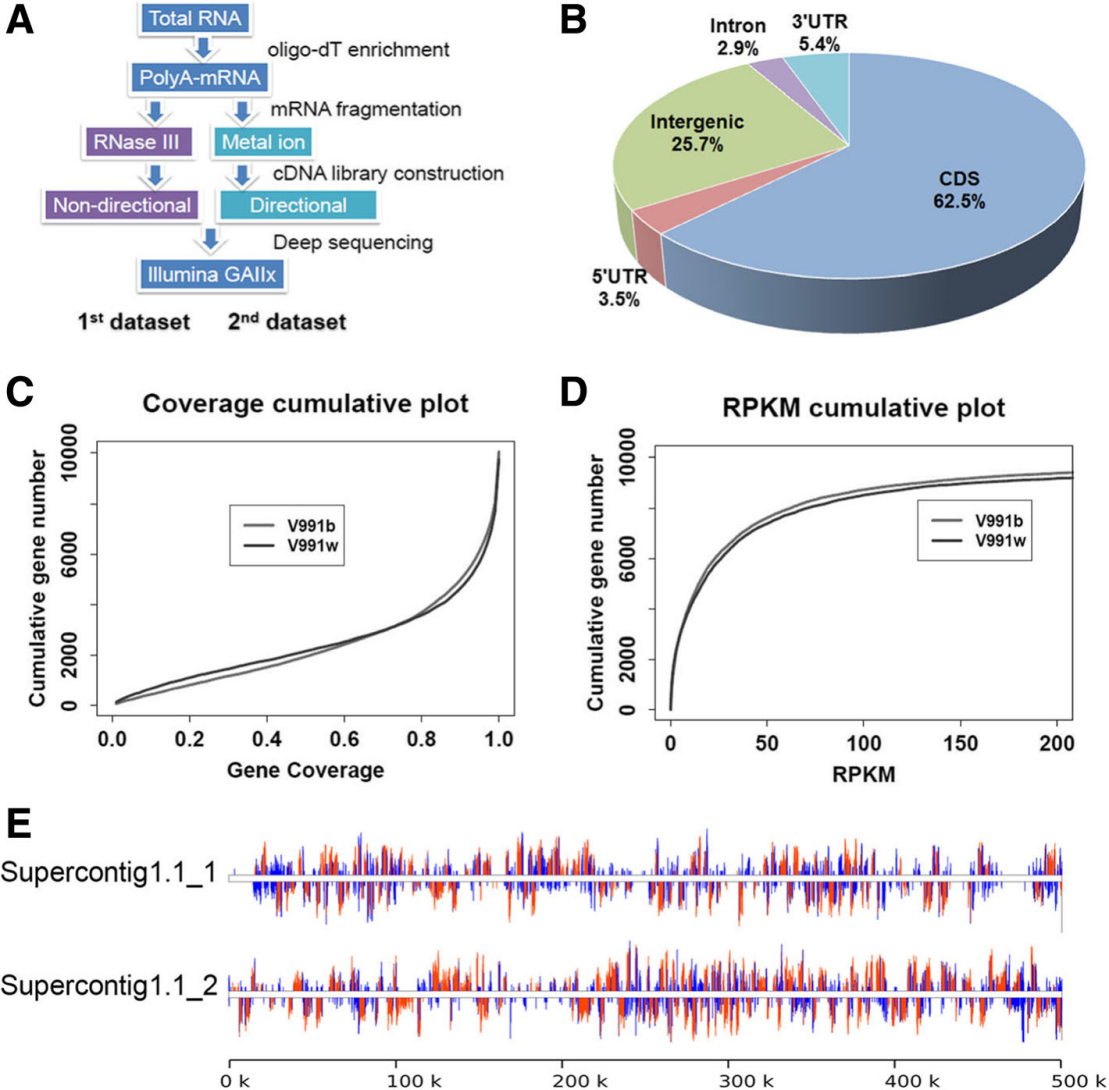
Expression profile of the annotated protein-coding genes in the *V. dahliae* genome. **a** The strategies used to generate the two sets of transcriptome data in this study. **b** Distribution of the mapped reads in different regions of the expressed genes. **c** RNA-seq reads coverage of all expressed genes. Base coverage of each mRNA by the mapped cDNA reads was first calculate, and the number of genes was then plotted against the coverage from 0 (no coverage) to 1 (100% coverage). **d** The expression level of all expressed genes represented by RPKM. RPKM stands for mapped Reads Per Kilo base of mRNA per Million reads. **e** Distribution of the mapped reads across the first 1000 kb of the supercontig 1 of the *V. dahliae* genome

In order to validate the results obtained using the RNase III fragmentation of polyA-mRNA, we constructed another set of cDNA libraries from the same *V. dahliae* strains using the ion fragmentation method instead (Fig. 1a). The total RNA was prepared from a different batch of microbe cultures to represent an independent biological repeat. A total of 15.7 and 13.9 millions of sequence reads were obtained for the V991b and V991w strains, with an overall mapping efficiency of 79.6 and 75.2%, respectively. In both cases, the unique mapped reads were about 98% of the total mapped, and about a quarter of which were mapped to the intergenic region (Fig. 1b). The intergenic transcripts were not from the specific genomic regions but rather genome-wide (Fig. 1e). The total reads mapped to the genic regions were 16.7 million for these two strains, which were 6-fold of the first set of data. Only 507 more genes (4.81%) were detected in the second one (Fig. 1c, Additional file 1: Figure S1B), suggesting an adequate sequence depth of the first one. The increased sequence depth substantially increased the gene coverage (Fig. 1c), while the RPKM values were substantially the same in both sets of data as expected (Fig. 1d, Additional file 1: Figure S1C), which further supported the confidence and quality of both datasets.

Interestingly, the second dataset showed a drastic increase in the sequence depth in the CDS region, and a sharp decrease in the 5′ UTR region. A less extent of decrease in the intronic and 3′ UTR regions was observed (Additional file 2: Table S1, Additional file 1: Figure S2D). These results supported a hypothesis that the 5′ UTR of this fungal pathogen are more enriched in local secondary structures.

### The prevalence of intron retention in the *V. dahliae* transcriptome

**Fig. 2:**
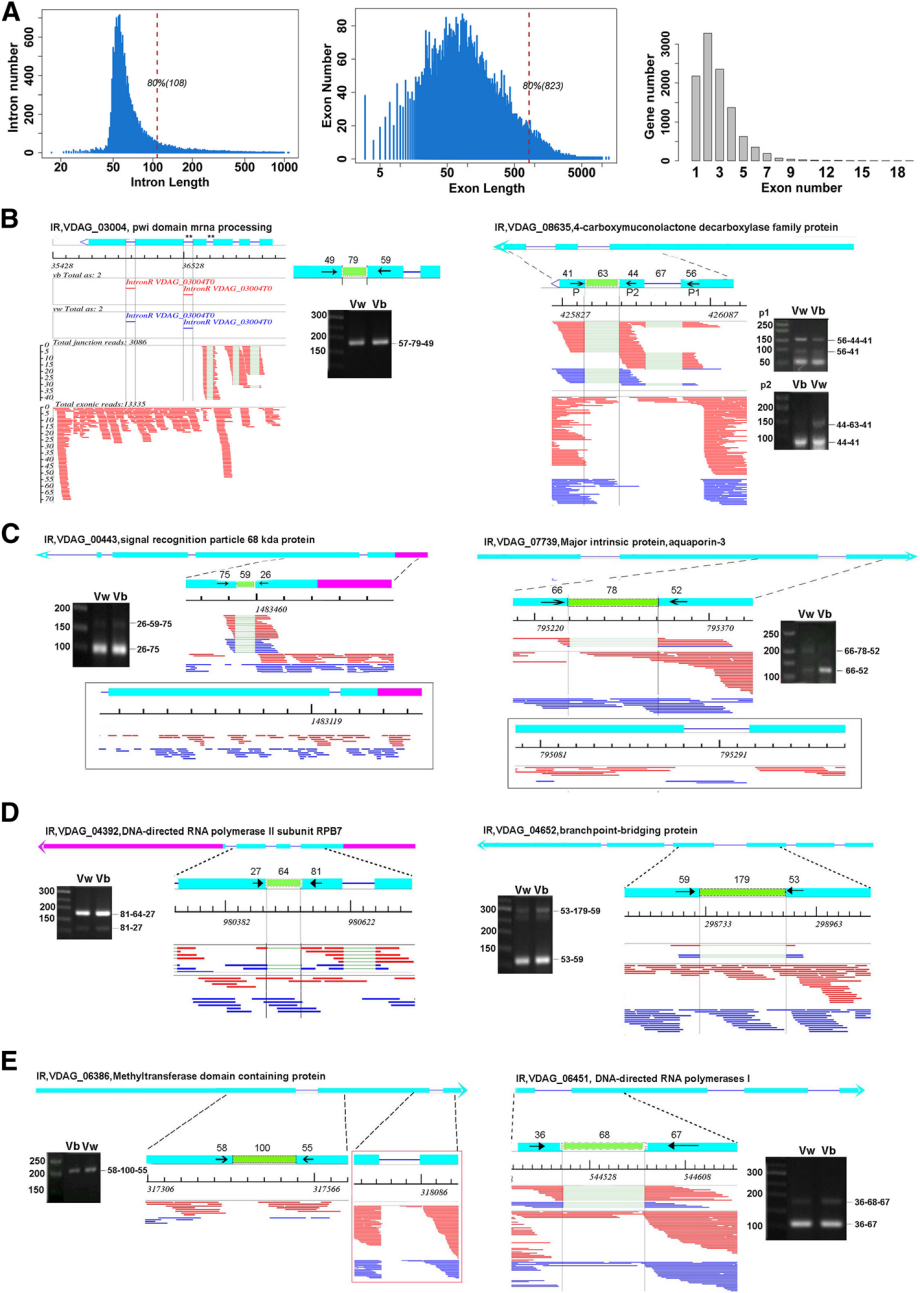
The prevalent regulation of mRNA output by intron retention (IR) in *V. dahliae*. **a** Structure features of *V. dahliae* genes. The statistics of the length distribution of all introns (*left*) and exons (*middle*), as well as the exon composition of each gene are shown. RT-PCR validation of IR events detected by the current iRAS algorithm from both datasets (**b**); from the 2nd but not the 1st dataset (**c**); from the 1st but not the 2nd (**d**); from neither (**e**). We developed an algorithm to visualize the alternative splicing events in each gene. For each gene, cDNA reads mapped to unique genomic locations were plotted and numbered, and the type of alternative splicing and the dataset in which the AS was detected were indicated. One full example was shown in (**b**, *left*), and the IR containing region used for RT-PCR is detailed above the electrophoresis gel. To simplify the presentation and emphasizing the read layout in the AS region, we show the structure of the gene containing the AS event at the top diagram with the known exons being boxed in *cyan*, the intron in *blue line* and the UTR regions in *purple boxes*. The AS is enlarged below with the positions of PCR primers being indicated as *black arrows*, the nucleotide length composing the spliced products being numbered, and the alternative exons boxed in *green*. The *black* scale (*horizontal axis*) and numbers below indicated the genomic location. The sequence reads spanning the splice junctions are shown below the scale. The *red* and *blue lines* indicate the reads from V991b and V991w, respectively. For junction reads, the unmatched intronic region are shown in *thinner green line*; the fully aligned reads are shown below. In a few cases, the read layouts from both dataset are shown, with the results from the 1st dataset being boxed (**c**). A representative electrophoresis gel of the RT-PCR validation result corresponding to each pair of primers is shown. Two batches of total RNAs were used for RT-PCR validation; the loading order of V991b and V991w was different

Survey of the *V. dahliae* genome reveals that 79.3% of the protein-coding genes (8359) contain at least one intron, predicting an important layer of genome regulation conducted by AS. The 19,150 introns cover 5.7% of the genome sequence. The average intron length was 100 bp and the intron-containing genes harbor an average of 2.3 introns. The annotated exons (29,685) have an overall average length of 530.7 bp, and intron-split exon of 476.4 bp (Fig. 2a). These features are quite different from the well-characterized human pathogen *Cryptococcus neoformans*, which has an average of 6.3 exons of 255 bp and 5.3 introns of 67 bp [17].

In order to globally assess the intron splicing feature of *V. dahliae* genome, we used the TopHat algorithm to identify all splice junctions (SJs) generated by splicing of primary transcripts when the pathogen was grown to the late log phase in vitro. In the first dataset, TopHat mapping of the combined sequence reads from both *V. dahliae* strains detected 81.84% of the annotated exons and 9343 (50.71%) of the known intron-associated SJ. The detection power was increased to 90.44% of the exons and to 58.30% (10,738) of the known SJs in the second dataset. Although the SJ detection efficiency depends on the sequence depth, the 6-fold increase in the sequence depth but slight increase in the detection of known SJ indicated that the sequence depth has reached a good saturation in this study. Therefore, the inability of detecting over 40% known SJs indicates that a large population of introns may not be spliced under the vegetative growth condition, which predicts a large number of intron retention events.

Our preliminary results showed that current available softwares were not suitable for detecting the fungal alternative splicing events (Data not shown). We developed an algorithm iRAS (intron Rentention associated AS event) to identify intron retention alternative splicing events. The algorithm had been improved through iterate matching of the iRAS results and the layout of mapped cDNA reads on each gene (Fig. 2b, left), with the final parameters being stated in Methods. The iRAS counts on the presence of intron-exon border read and the mean base depth of the retained intron being at least 20% of the flanking exon. A total of 2015 and 3575 of IR events were detected from the first and second *V. dahliae* transcriptome datasets; 1558 of them were overlapped indicative of their reliability (Table 1, Additional files 3 and 4).

**Table 1:** Statistics of alternative splicing (AS) events and genes in *V. dahliae* transcriptomes

	AS type	V991w	V991b	All	Total	OL	Frequency	Genes	OL
AS events involving one alternative splice site									
	**A3SS**	**221/372**	**230/378**	**308/466**	**582**	**256**	**11.63%**	**515**	**243**
	A3SS&ES	3/5	2/9	3/5	12	3	0.14%	11	3
	**A5SS**	**150/221**	**139/232**	**211/294**	**379**	**163**	**7.40%**	**357**	**161**
	A5SS&ES	6/11	4/9	9/12	15	7	0.32%	13	7
	5pMXE	15/18	11/16	17/20	23	14	0.64%	23	14
	3pMXE	1/2	0/2	1/2	2	1	0.05%	2	1
	MXE	2/1	1/0	3/1	3	1	0.05%	3	1
	ES	9/12	5/13	10/15	18	9	0.42%	18	9
	**Cassette Exon**	**35/61**	**47/62**	**62/89**	**104**	**49**	**2.23%**	**98**	**50**
					1138	503	22.85%	1040	489
Intron retention									
	IntronR	1305/2560	1247/2568	1935/3236	4033	1558	70.79%	2955	1339
AS events involving a pair of novel splice sites									
	Cassette Intron	64/207	62/223	106/299	325	100	4.54%	318	100
	Intronic intron	32/73	39/73	58/108	138	40	1.82%	127	40
	Total	1843/3543	1787/3577	2723/4547	5634	2201	100.00%	4440	1968

The results of both datasets were shown in pair, in the 1st/2nd format. The number of the events or genes overlapped (OL) between the datasets is indicated

Three major types of non-IR as events were bolded

We validated over a dozen of intron retention events using RT-PCR analysis (Fig. 2b-e). These events represent four different classes of IR events. The first class includes those detected in both datasets, which are the 1558 overlapped IR events shown in Table 1 (Fig. 2b). The second represents those detected only in the 2nd dataset, but not the 1st, and the third represents those only in the 1st (Fig. 2c and d). The fourth class represents those identified by less stringent criteria in the previous version of iRAS, but not the final version presented here (Fig. 2e). We demonstrated that all these classes were positive for RT-PCR validation. These results suggest that intron retention could be much more prevalent than shown in Table 1. It is also noteworthy that some of these IR events differed in their intron inclusion frequency, which suggested AS would contributed to the different characterize of the two strains.

Intron retention could be resulted from polyadenylation of the primary transcripts without splicing of the existing intron(s), or specific retention of some intron(s) but not the other in a gene. Statistical analysis showed that for the genes containing two or more introns, most IR events were intron-specific (Table 2). Intron retention could alter the protein coding potential of a gene by breaking the coding sequence, and may also interfering the mRNA stability by introducing premature termination codons (PTCs). Statistical analysis shows that 474 of the non-last-intron IR events could result in PTCs (Table 2, Additional file 5); the point will be discussed below.

**Table 2:** Intron retention (IR) and premature termination codons (PTCs) in *V. dahliae* genome

Intron per gene	Intron-containing genes				PTCs
	All	Non-specific IR	Specific IR	No IR	
1	3228	732 (22.68%)		2496 (77.32%)	
2	2396	216 (9.02%)	665 (27.75%)	1515 (63.23%)	201
3+	2800	60 (2.14%)	1282 (45.79%)	1458 (52.07%)	273

### Hundreds of novel intron types involving a pair of new splice sites: cassette intron and intronic intron in the *V. dahliae* transcriptome

**Fig. 3:**
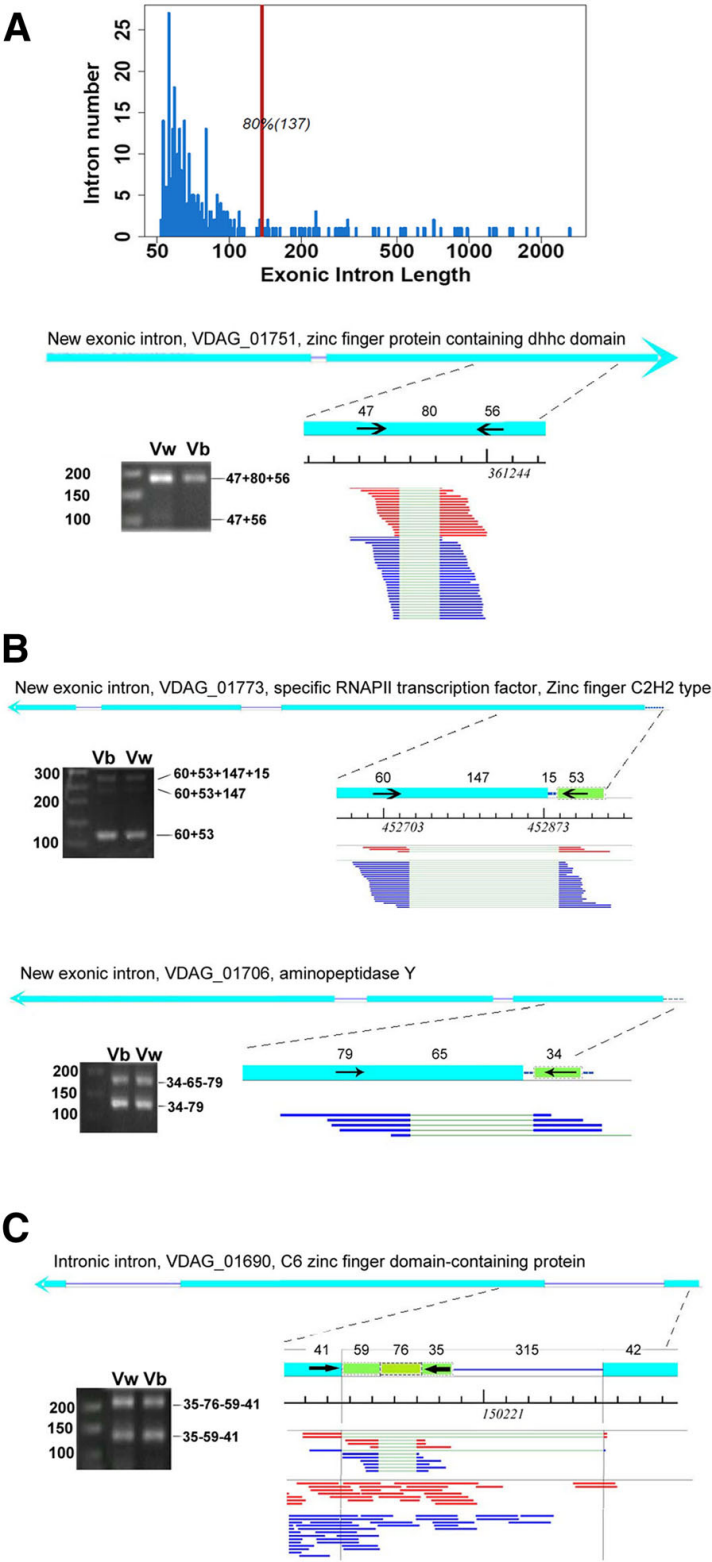
Cassette intron and intronic intron involving a pair of novel splice sites. **a** The newly identified exonic introns. The statistics of their length distribution (*left*); RT-PCR validation of one representative of these introns (*right*). **b** Exonic introns involving extended UTRs. **c** Intronic intron. Labels are similar to those in Fig. 2

The long-exon feature of the current *V. dahliae* genome could be specific for *V. dahliae* or resulted from an insufficient annotation of introns. Searching for the novel introns residing in the annotated exons, over three hundreds of new spliced introns were identified from the two datasets, which did not appreciably alter the long-exon feature (Table 1, Additional file 6). The length distribution of these new introns were similar to the known (Fig. 3a, top), and many spliced completely (Fig. 3a, bottom). Meanwhile, nearly two thirds (179) of the exonic introns were also identified as IR events, demonstrating that splicing of these introns are alternative. When we set the reading frame of this exonic intron containing gene from the annotated start codon and through all upstream exons followed by the new intron, stop codons were only present in two of these introns, which thus excluded the possibility that these introns resulted from mis-annotation of the *V. dahliae* genes. Therefore, we conclude that these exonic introns are intrinsically part of the coding sequence, and could alternatively act as non-coding introns which we named as “Cassette Intron”, a counterpart of “Cassette Exon” standing for the intronic coding sequence. Apparently, splicing of cassette intron results in mRNA coding for a shorter protein than that of the unspliced. In addition, it is noteworthy that the nucleotide length of about 45% of these introns is not multiple of three, predicting that the downstream coding frame will be altered when the intron is spliced. Therefore, cassette intron represents a new type of alternative splicing event that profoundly alter the mRNA output from fungal genes.

When we investigated the cDNA reads layout on the genome, we found that there were also cases of exonic intron with most of the intron residing in the known exon, and a small fraction extending into the 5′ UTR region (Fig. 3b). This type of splicing resulted in 5′ UTR-containing mRNA isoforms having truncated 5′ coding sequences. We validated two of these special exonic intron in genes encoding zinc finger-type RNA polymerase II transcription factor and aminopeptidase Y, and found in both cases, alternative mRNA isoforms were generated, one containing the intron and one lacking (Fig. 3b). These results suggest that exonic intron may play an important role in regulating the 5′ coding sequences of fungal genes.

In addition, 141 intronic introns were detected from the transcriptome as well (Table 1 and Fig. 3c). From the structure view, the intronic introns are nested introns. From the view of splicing consequence, the coding potential of the new mRNA isoform resulted from the usage of intronic intron splice site is altered.

### Over one thousand of *V. dahliae* alternative splicing events involve in at least one known splice site and an alternative splice junction

We then analyzed the more common AS events involving alternative splice junction, using the annotated known splice junctions as the reference. TopHat novel SJs includes all potential SJs other than the annotated SJs, which could be produced from the alternative splicing of a pre-mRNA involving one or two novel splice sites, or splicing of the known splice sites in an alternative way. Our first and second datasets detected 5315 and 14,575 novel SJs, respectively. The confidence of these SJs was validated by plotting the number of reads supporting each of the identified SJs in the two *V. dahliae* strain. A large fraction of the novel SJs was supported by 2 or more reads (Additional file 1: Figure S3A and B).

#### Improved annotation of the 5′ and 3′ UTR of the V. dahliae genome

**Fig. 4:**
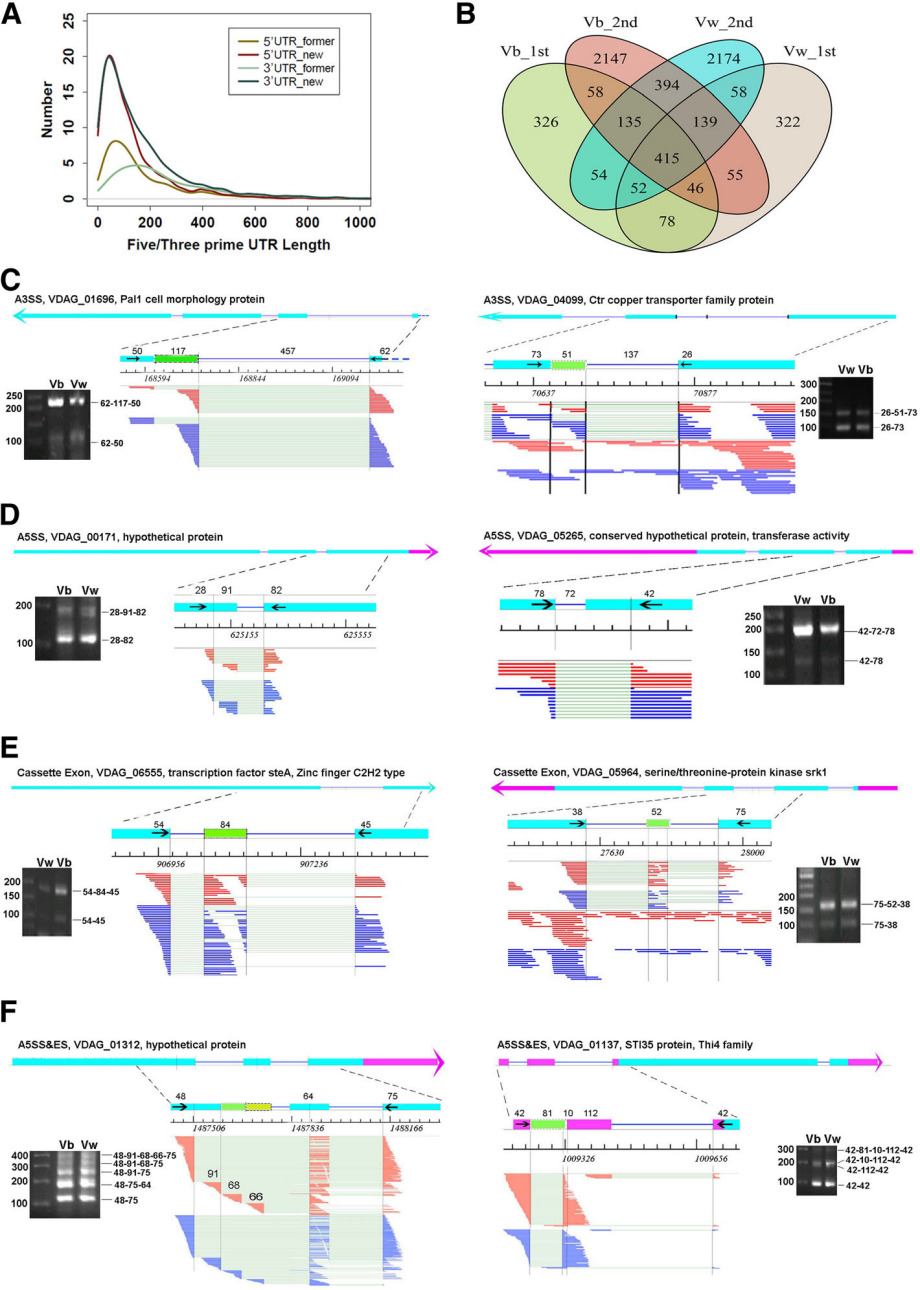
The complex alternative splicing patterns. **a** Re-annotation of the 5′ and 3′ UTRs of *V. dahliae*. **b** Overlap of the genic novel SJs among the transcriptomes of *V. dahliae* strains V991b and V991w of both datasets. **c-e** Results of RT-PCR validation of typical alternative splicing events involving one alternative splice sites: alternative 3′ splice sites (A3SS); alternative 5′ splice site (A5SS), and Cassette Exon. **f** The combinatory alternative splicing involving both A5SS and exon skipping. Labels are similar to those in Fig. 2

We noticed that many novel SJs were located in the intergenic regions (Additional file 1: Figure S3C). It could be possible that some intergenic SJs were derived from the unannoted UTR regions. We therefore re-annotated the UTR regions according to the expression information of the second transcriptome data containing RNA direction information. The new annotation resulted in 1591 and 2430 of new 5′ and 3′ UTRs, respectively; it also extended 146 and 78 of the existed 5′ UTRs and 3′ UTRs, respectively. This information was added to the previously published GFF (Generic Feature Format) file and created an improved GFF used for further study (Additional file 7). *V. dahliae* genes with UTR annotations were increased from 2422 to 5803. Interestingly, although the previously annotated 3′ UTRs are generally longer than 5′ UTRs, most expressed UTRs are within 100-nt, which is similar to the intron length (Fig. 4a).

The new annotation information allowed us to obtain 591 and 1083 genic novel SJs shared by both *V. dahliae* strains from the 1st and 2nd transcriptome dataset, respectively. A total of 415 novel SJs were detected in all four *V. dahliae* transcriptomes (Fig. 4b). These results suggested that fungus cells may actively utilize alternative splicing events involving alternative splice sites to exert its genome regulation task.

#### aJAS algorithm identified over one thousand alternative splicing events

We developed an algorithms aJAS (alternative-splice-Junction associated AS events) (Additional file 1: Methods) to identify the qualified alternative splicing events from the genic TopHat novel SJs. The aJAS events were classed into seven canonical types including alternative 5′ splice site (A5SS), alternative 3′ splice site (A3SS), cassette-exon (CE), exon skipping (ES), mutually exclusive exons (MXE), mutually exclusive 5′ UTRs (5pMXE) and mutually exclusive 3′ UTRs (3pMXE); and into two combinatory types including the combination of alternative 3′ splice site and exon skipping (A3SS&ES), and the combination of alternative 5′ splice site and exon skipping (A5SS&ES) (Table 1). A total of 657 and 984 of aJAS events were detected from the 1st and 2nd transcriptome datasets, respectively. Strikingly, 503 of them were identified from both datasets, supporting the confidence of aJAS events (Table 1, Additional files 8 and 9). The two largest types of aJAS events were alternative 3′ and 5′ splice site, representing ~50 and ~35% of the total aJAS events, respectively. About 10% of the aJAS are cassette exon, i.e. alternative exons residing in annotated introns. As detailed below, these aJAS events were highly validatable by RT-PCR.

It is noteworthy that 227 of the aJAS events were detected in all four transcriptome datasets (Additional file 1: Figure S4B). Compared to the total number of genic TopHat SJs, over a half of them were identified as true alternative splicing events, which indicate that TopHat novel SJs are reliable sources for identification of alternative splicing events in fungal transcriptome.

#### Most alternative spliced isoforms were validated by RT-PCR

We picked up 38 aJAS events for RT-PCR validation, 30 of them were validated by RT-PCR, which includes the most abundant A3SS, A5SS and Cassette Exon (Fig. 4c-e), as well as the less frequent 5pMXE, and 3pMXE, as well as exon skipping (ES) (Additional file 1: Figure S5). There were two major reasons for the failure of detecting the expected bands corresponding to aJAS events, as well as those to the iRAS events described above. First, the expression level of the related mRNA isoform was too low to be detected by RT-PCR analysis, as reflected by the low number of supporting cDNA reads. In addition, the potential sequence variation between the reference genome and the genome of the experimental strains at the primer regions might result in no PCR product.

Among the validated alternative splicing events, there were quite a number of them detected in the 2nd dataset but not the 1st due to the lower sequence depth. These included the two A5SS events shown in Fig. 4f, 3pMXE (VDAG_07278) and ES (VDAG_05517) (Additional file 1: Figure S5). Moreover, the 5pMXE (VDAG_01709) validated by RT-PCR was detected by a previous version of aJAS, but not by the current one (Additional file 1: Figure S5A). These results together demonstrate the reliability of aJAS alternative splicing events shown in this study and the large impact of aJAS events in regulating the mRNA output of the fungal pathogen.

#### The combinatory alternative splicing contributes to the mRNA complexity in V. dahliae

Interestingly, during the course of developing the aJAS algorithm, we found the presence of more complex alternative splicing events involving the combination of ES with A5SS (Fig. 4f), or with A3SS (Additional file 1: Figure S6A). We therefore included the AS patterns in the current aJAS algorithm, and identified a total of 27 of these AS events. These combinatory events were highly validatable. It is surprising to find that one *Verticillium* gene (VDAG_01312) applied four different 5′ splice sites and two different 3′ donor sites to produce up to five mature mRNAs (Fig. 4f), which probably represent one of the most efficient alternative splicing case reported so far. This gene encodes a hypothetical protein, and such a splicing pattern is conserved in both *V. dahliae* strain, indicating a house keeping function of this splicing pattern.

During the validation process, we also found some other combinatory alternative splicing events, including the combination of Cassette Exon with A5SS and with IR (Additional file 1: Figure S6B and C).

### Alternative splicing does not appreciably contribute to the fungal mRNA levels

The alternative spliced mRNAs containing premature termination codons (PTC) within an appropriate distance to the last exon-exon junction tend to be linked to the nonsense mediated decay (NMD) machinery in mammals [32]. It has been estimated that one third of the naturally occurring, alternatively spliced mRNAs are targets of NMD [33, 34]. The link between alternative splicing and NMD is emerging recently in plants [35]. Most of the AS genes harbor only one alternative splicing event in *V. dahliae*, generating a simple mRNA isoform profile. Among 2955 genes containing intron-retention AS events, we found 474 containing PTCs that are potential targets of NMD (Table 2). A total of 146 of these genes were among the 2789 differentially expressed genes (DEG) in the 1st and/or the 2nd datasets, which shows no statistical significance over the non-PTC containing genes (data not shown).

**Fig. 5:**
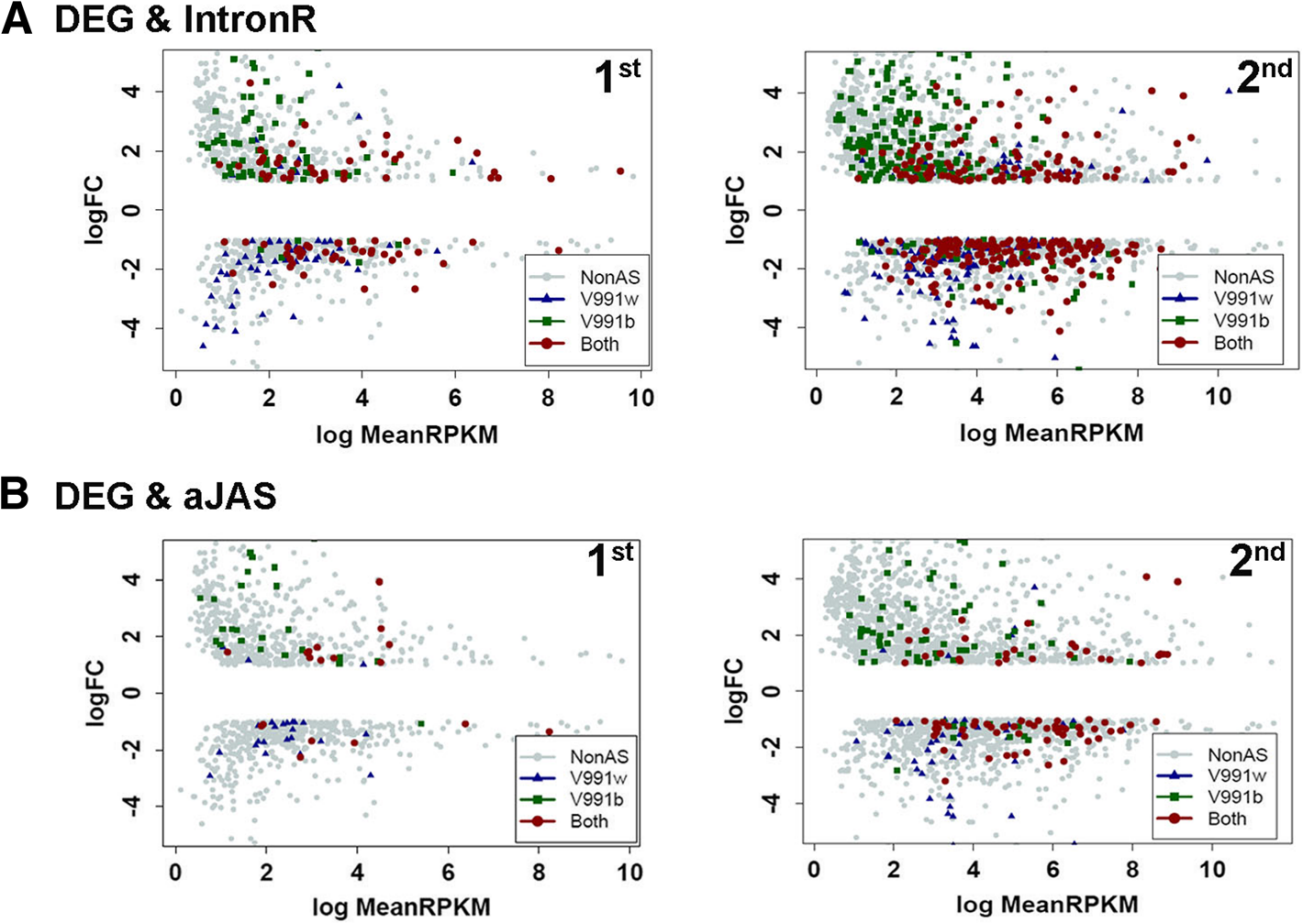
The correlation between alternative splicing and differential gene expression. The correlation between differentially expressed genes and intron retention events (**a**); and alternative splicing involving one alternative splice site (**b**). Among the differentially expressed genes between V991b and V991w identified in each of the two datasets, those containing at least one of the indicated alternative splicing events are indicated. Left-1st dataset, Right-2nd dataset. The AS events detected in one strain but not the other, or in both strains are indicated by different symbols. *Horizontal axis* (logMeanRPKM) indicates the express level of each gene; *Vertical axis* (logFC) indicates the fold change of a gene expression level between V991b and V991w. *Each dot* represents one differentially expressed gene

To address the question, we asked the association of AS event detection in the differentially expressed genes between the two *V. dahliae* strains. As clearly demonstrated in Fig. 5, when an AS event was overlapped with DEG, they were predominately detected in the *V. dahliae* strain showing higher expression level of the gene, indicating that the association is strongly depended on the transcription discrepancy. The AS genes showing the reverse expression pattern indicated PTC-associated genes were not significantly influenced by NMD pathway. Overall, we concluded that the steady-state level of polyadenylated mRNA in fungal pathogen dominantly reflects the transcriptional regulation, while AS regulation could exert its function primarily by regulating the mRNA and protein isoforms.

### Alternative splicing is predicted to extensively regulate fungal functions

As shown above, a total of 5634 AS events were detected from the first and second transcriptome datasets of the two *V. dahliae* strains. These events were well overlapped (Additional file 1: Figure S4A) and quite reliable. They were located in over four thousand genes. The majority of these genes contain one detected AS event. The genes from the 1st dataset were mapped to 2467 biological function terms of GO, including 877 of biological process terms, 1132 of molecular function terms and 457 of cellular components. The high node scores of AS genes included many terms of biological processes, cellular components and molecular function, suggesting the AS mechanism regulates molecular activities and biological functions in fungi.

**Fig. 6:**
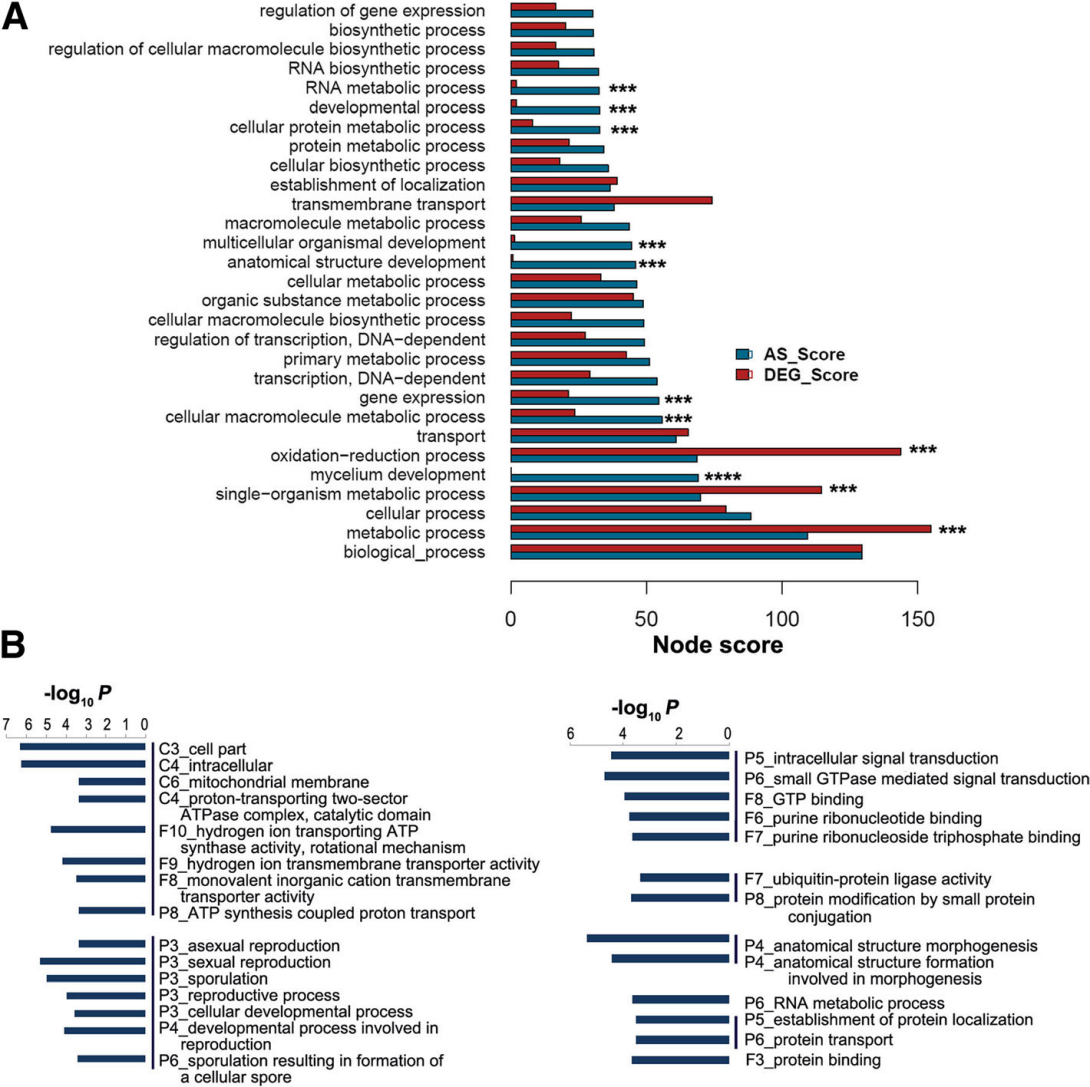
Alternative splicing regulation of the biological processes of *V. dahliae*. **a** Representative of biological functions controlled by alternative splicing in the plant pathogen. All the biological process terms with a node score larger than 30 were selected for presentation, and the node scores of corresponding terms were also plotted. Asterisks indicate the terms preferentially controlled either by transcriptional regulation or alternative splicing. **b** GO analysis of the alternative splicing genes by using the whole genome as a background. To fit the size, the plot was split into two parts (*left* and *right*)

Comparison of AS gene terms with high node scores with their counterpart DEG gene terms revealed a substantial difference (Fig. 6a, Additional file 1: Figure S7 and S8). Genes under alternative splicing regulation are enriched in mycelium development, multicellular organism development and anatomical structure development. Meanwhile, AS genes are more enriched in gene expression, protein and RNA metabolic processes than DEG genes.

When the GO terms of the whole genome were used as a background, AS genes were readily clustered into several distinct biological pathways (Fig. 6b). These include the mitochondrial membrane-proton transport-ATP synthesis pathway, anatomical structure morphogenesis, sexual reproduction-sporulation-development pathway, small GTPase mediated signal transduction pathway, protein transport-localization pathway and RNA metabolic pathway. These data collectively support a concept that alternative splicing regulation is wired into the fungal genome expression and regulation network and potentially controls most biological activity, process and function of *V. dahliae*, with a strong preference to control the regulatory biological functions highly related to the life cycle of the pathogen after it infects the plant host.

## Discussion

Alternative splicing is a key mechanism for protein diversity from a given gene and for proteome complexity of a given genome under a specific condition. Analysis of the 23,000 full transcripts of the human pathogen *C. neoformans* resulted in the identification of 277 alternatively spliced genes (4.2% of the transcriptome) [17], whereas the similar approach has resulted in the discovery of about one or two hundreds of alternative splicing events in the plant pathogens *U. maydis* and *Magnaporthe grisea* [16, 36]. Deep sequencing of the transcriptome of the plant pathogens *Aspergillus oryzae* and *Fusarium graminearum* revealed over a thousand and two hundreds of such events, respectively [14, 18]. These two deep sequencing studies both showed that about 90% of the alternative splicing events are resulted from intron retention; the later study validated several AS events, including three cases of intron retention and one case of alternative 3′ splice site choice (A3SS) and a couple of them under the developmental regulation.

The transcriptome profile of *V. dahliae* in different growth conditions were investigated previously, and the alternative splicing was studied by using SplicingViewer [37]. The study showed that over 95% of AS events were intron retention, only ~1% of AS events were exon skipping, A3SS or A5SS events in *V. dahliae*, respectively. Though three IR events were supported by RT-PCR, other forms of AS events were not validated. Herein, our study developed specific algorithms to accurately reveal the alternative splicing events in the fungal pathogen *V. dahliae*. Four different transcriptome data were generated by deep sequencing, being used to better annotate the 5′ and 3′ UTRs of the protein coding genes and for identification of the previously unannotated introns in all genic regions. Alternative splicing by intron retention and those associated with novel/alternative splice junctions has been separately classified. And the accuracy of the characterized alternative splicing events was manually checked by comparing to the cDNA read layout figures generated for each gene; the results were used to revise and improve the algorithms. The high efficiency of RT-PCR validation of the AS events further confirmed the accuracy of the algorithms.

This study has shown that about 50% of the multi-exonic genes of *V. dahliae* genes are under alternative splicing regulation, including 4033 intron retention, 582 alternative 3′ SS, 379 alternative 5′ SS, and 104 cassette exon and tens of other complex alternative splicing events. Though IR is still the major AS events in *V. dahliae*, the ratio of other types of AS events reaches up to ~30%, which emphasized a more comprehensive and diversified mechanism of AS regulation involved in transcriptome diversity. Moreover, over three hundreds of exonic introns have been identified, which were classified as an important new type of alternative splicing. For the first time, we reported the presence of combinatory alternative splicing patterns in *V. dahliae*, further underling the phylogenetic importance of fungus in developing alternative splicing mechanism.

## Conclusion

The study has shown the prevalent roles of alternative splicing in shaping the transcriptome and proteome complexity of the lower eukaryotes. Taken together the identification of dozens of complicate alternative splicing patterns involving the combination of two different alternative splicing events in *V. dahliae*, we conclude that the wilt pathogen *V. dahliae* has an important phylogenetic role during the development of alternative splicing mechanism. Consistently, the AS frequency and distribution of different AS patterns in *V. dahliae* transcriptome is strikingly similar to that of *Arabidopsis* and rice detected by comprehensive EST analysis or transcriptome sequencing, with the preferred patterns in order of IR, A3SS (acceptor), A5SS (donor) and cassette exon/exon skipping [9, 38]. Functional clustering of the genes under alternative splicing regulation showed that this layer of genome regulation play critical roles in fungal development and reproduction, signal transduction, mitochondrial functions and many other biological functions including both the transcriptional and post-transcriptional processes.

## Methods

### *V. dahliae* strains

The V991w and V991b strains were kindly provided by Prof. Xianlong Zhan (Huangzhong Agricultural University, China), both were isolated from the cotton field [39].

### cDNA preparation for high-throughput Illumina sequencing

The total RNA from the late-log phase culture of each strain was extracted using RNAisoTM Plus (TaKaRa) and then treated with RNase-free DNase I (TaKaRa) for 45 min according to the manufacturer’s protocols. Two batches of fungal cultures were prepared for total RNA preparations. The cDNA libraries for the first batch of fungal cultures were prepared from polyadenylated mRNA/ncRNA isolated using oligo-dT beads (NEB) and fragmented by RNase III (NEB). PCR amplification was used to enrich the adapter-attached cDNA library using primers complementary to the ends of the adapters for single-end sequencing (Illumina). The library products were sequenced at 80-nt length using the Illumina Genome Analyzer IIx. For the dataset generated by RNase III fragmentation of mRNA, only the raw reads with their resulted clean reads mapped onto the gene regions of *V. dahliae* genome were analyzed in this study and deposited. The cDNA libraries for the second batch of fungal cultures were prepared from polyadenylated mRNA/ncRNA isolated using oligo-dT beads (Invitrogen) and ion fragmented at 95 °C followed by end repair and 5′ adaptor ligation. Then the reverse transcription was performed with RT primer harboring 3′ adaptor sequence and randomized hexamer. The cDNA libraries were sequence the same as the first.

### Reads mapping and primary analysis

The *V. dahliae* genome sequence and gene annotation were downloaded from Broad Institute (http://www.broadinstitute.org/). Sequence reads passed quality filter (the reads containing less than 2 Ns) and removed adapter sequence from ends were aligned to the genome using TopHat tool allowing the maximum of two mismatches [40]. The reads mapped onto the annotated gene regions were subjected to the statistical analysis. The expression level for each gene was normalized by the number of reads per kilo base per million mapped reads (RPKM).

### Alternative splicing events in *Verticillium* transcriptome

In this study, the known/model splice junctions (SJs) was defined by joining splice sites at the exon-intron boundaries of all annotated introns in the recently published genome [28], generating a total of 19,150 such model/known SJs. All other SJs involving only one or none of the known splice site were considered as novel junction. SJs in the transcriptome of *V. dahliae* supported by cDNA reads were identified using TopHat tool [40]. Junction reads were required to have at least 8-nt mapped on each of the adjacent exons. The junctions located inside of the coordinates of annotated genes were regarded as genic SJs. All the genic SJs were classified into one of the nine types of AS events. Seven of canonical AS events were skipped exons (ES), cassette exon (CE), alternative 5′-splice sites (A5SS), alternative 3′-splice sites (A3SS), mutually exclusive exons (MXE), alternative first exons (AFE or 5′ MXE) and alternative last exons (ALE or 3′ MXE), according to the models described previously [6]. Algorithm aJAS is based on a given gene model and calculates sequence reads supporting each distinct composite SJs associated with a specific novel SJ. The novel SJs containing at least two support reads were selected for analysis. To be a qualified candidate aJAS event, the ratio of reads supporting the novel/alternative SJs to the total was at least 15%.

Retained intron or intron retention (RI or IR) is caused by reduced usage of the candidate splice sites, which cannot be predicted effectively by considering splice junctions. This class of alternative splicing event was identified according to the border reads spanning exon-intron junction and the mean of local reads depth. Four criteria have to be met to be considered as a RI event. (1) The mean base depth in the candidate intron is at least 20% of the flanking exon; (2) The sum of the intronic depth is greater than 100; (3) border reads at either the 5′ or 3′ splice site of the candidate must be present; (4) no other type of AS event could be identified.

### Functional enrichment analysis

Gene ontology classification database with Blast2go package was used to perform the functional cluster of the differentially expressed or spliced genes. The method perform Fisher exact test with robust false discovery rate (FDR) correction to obtain an adjusted *P*-value between certain tested gene groups and the whole annotation [41].

### Validation of AS events

RT-PCR (Real-time Polymerase Chain Reaction) was used to validate the alternative splicing events. Total RNA was prepared from the first batch of *V. dahliae* cultures, which represented a preparation different from that for transcriptome sequencing. Primers used in this study were listed in the Additional file 2: Table S1.

### Data access

Illumina short read sequences (80 nt for the 1st and 73 nt for the 2nd datasets) generated in this study have been deposited in NCBIs Gene Expression Omnibus (GEO, http://www.ncbi.nlm.nih.gov/geo/) GEO under the accession number GSE45936.

## Additional files

Additional file 1: Figure S1. Expression profile of the annotated protein-coding genes in the *V. dahliae* genome revealed by the 1st dataset. **Figure S2.** The *V. dahliae* non-coding regions could be enriched in secondary structures. **Figure S3.** Statistics of the TopHat junction reads. **Figure S4.** Overlap of the all AS genes. **Figure S5.** Mutually exclusive exons. **Figure S6.** Combinatory alternative splicing patterns for *V. dahliae* pre-mRNA alternative splicing. **Figure S7.** Representative of biological functions controlled by alternative splicing in the plant pathogen. **Figure S8.** Representative of biological functions controlled by alternative splicing in the plant pathogen. (DOCX 1433 kb)
Additional file 2: Table S1. The genic distribution of two sets of transcriptome sequencing reads from the two *V. dahliae* strains. **Table S2.** Statistics of the expressed exons, introns and splice junctions (SJ) in the vegetative grown V. dahliae (1st dataset). **Table S3.** PCR Primers used in this study. (DOCX 32 kb)
Additional file 3: Table S4. IntronR-1st dataset. (XLSX 125 kb)
Additional file 4: Table S5. IntronR-2nd dataset. (XLSX 203 kb)
Additional file 5: Table S6. Potential IR-induced PTCs for non-sense mediated decay. (XLSX 36 kb)
Additional file 6: Table S7. Exonic Intron-1st & 2nd datasets. (XLSX 43 kb)
Additional file 7: GFF annotation. *V. dahliae* genome annotation_additional UTRs and Exonic intron_for AS analysis. (ZIP 1332 kb)
Additional file 8: Table S8. aJAS & Intronic intron-1st dataset. (XLSX 52 kb)
Additional file 9: Table S9. aJAS & Intronic intron-2nd dataset. (XLSX 74 kb)

## Abbreviations

3pMXE: Mutually exclusive 3′ UTRs
5pMXE: Mutually exclusive 5′ UTRs
A3SS: Alternative 3′ splice site
A3SS&ES: Alternative 3′ splice site and exon skipping
A5SS: Alternative 5′ splice site
A5SS&ES: Alternative 5′ splice site and exon skipping
aJAS: Alternative splice Junction associated AS events
AS: Alternative splicing
CE: Cassette exon
DEGs: Differentially expressed genes
ES: Exon skipping
FDR: False discovery rate
iRAS: Intron Rentention associated AS event
MXE: mutually exclusive exons
RPKM: Reads per kilo base per million mapped reads
RT-PCR: Real-time Polymerase Chain Reaction
SJs: Splice junctions
*V. dahlia*: *Verticillium dahliae*

## References

[1] Kahvejian A, Quackenbush J, Thompson JF (2008). What would you do if you could sequence everything?. Nat Biotechnol.

[2] Voineagu I, Wang X, Johnston P, Lowe JK, Tian Y, Horvath S, Mill J, Cantor RM, Blencowe BJ, Geschwind DH (2011). Transcriptomic analysis of autistic brain reveals convergent molecular pathology. Nature.

[3] Xue Y, Zhou Y, Wu T, Zhu T, Ji X, Kwon YS, Zhang C, Yeo G, Black DL, Sun H (2009). Genome-wide analysis of PTB-RNA interactions reveals a strategy used by the general splicing repressor to modulate exon inclusion or skipping. Mol Cell.

[4] Xiao R, Tang P, Yang B, Huang J, Zhou Y, Shao C, Li H, Sun H, Zhang Y, Fu XD (2012). Nuclear matrix factor hnRNP U/SAF-A exerts a global control of alternative splicing by regulating U2 snRNP maturation. Mol Cell.

[5] Xue Y, Ouyang K, Huang J, Zhou Y, Ouyang H, Li H, Wang G, Wu Q, Wei C, Bi Y (2013). Direct conversion of fibroblasts to neurons by reprogramming PTB-regulated microRNA circuits. Cell.

[6] Wang ET, Sandberg R, Luo S, Khrebtukova I, Zhang L, Mayr C, Kingsmore SF, Schroth GP, Burge CB (2008). Alternative isoform regulation in human tissue transcriptomes. Nature.

[7] Filichkin SA, Priest HD, Givan SA, Shen R, Bryant DW, Fox SE, Wong WK, Mockler TC (2010). Genome-wide mapping of alternative splicing in *Arabidopsis thaliana*. Genome Res.

[8] Zhang G, Guo G, Hu X, Zhang Y, Li Q, Li R, Zhuang R, Lu Z, He Z, Fang X (2010). Deep RNA sequencing at single base-pair resolution reveals high complexity of the rice transcriptome. Genome Res.

[9] Marquez Y, Brown JW, Simpson C, Barta A, Kalyna M (2012). Transcriptome survey reveals increased complexity of the alternative splicing landscape in *Arabidopsis*. Genome Res.

[10] Li Q, Xiao G, Zhu YX (2014). Single-nucleotide resolution mapping of the *Gossypium raimondii* transcriptome reveals a new mechanism for alternative splicing of introns. Mol Plant.

[11] Shen Y, Zhou Z, Wang Z, Li W, Fang C, Wu M, Ma Y, Liu T, Kong LA, Peng DL (2014). Global dissection of alternative splicing in paleopolyploid soybean. Plant Cell.

[12] Thatcher SR, Zhou W, Leonard A, Wang BB, Beatty M, Zastrow-Hayes G, Zhao X, Baumgarten A, Li B (2014). Genome-wide analysis of alternative splicing in *Zea mays*: landscape and genetic regulation. Plant Cell.

[13] Gonzalez-Hilarion S, Paulet D, Lee KT, Hon CC, Lechat P, Mogensen E, Moyrand F, Proux C, Barboux R, Bussotti G (2016). Intron retention-dependent gene regulation in *Cryptococcus neoformans*. Sci Rep.

[14] Wang B, Guo G, Wang C, Lin Y, Wang X, Zhao M, Guo Y, He M, Zhang Y, Pan L (2010). Survey of the transcriptome of *Aspergillus oryzae* via massively parallel mRNA sequencing. Nucleic Acids Res.

[15] Xie BB, Li D, Shi WL, Qin QL, Wang XW, Rong JC, Sun CY, Huang F, Zhang XY, Dong XW (2015). Deep RNA sequencing reveals a high frequency of alternative splicing events in the fungus *Trichoderma longibrachiatum*. BMC Genomics.

[16] Ho EC, Cahill MJ, Saville BJ (2007). Gene discovery and transcript analyses in the corn smut pathogen *Ustilago maydis*: expressed sequence tag and genome sequence comparison. BMC Genomics.

[17] Loftus BJ, Fung E, Roncaglia P, Rowley D, Amedeo P, Bruno D, Vamathevan J, Miranda M, Anderson IJ, Fraser JA (2005). The genome of the basidiomycetous yeast and human pathogen *Cryptococcus neoformans*. Science.

[18] Zhao C, Waalwijk C, de Wit PJ, Tang D, van der Lee T (2013). RNA-Seq analysis reveals new gene models and alternative splicing in the fungal pathogen *Fusarium graminearum*. BMC Genomics.

[19] Li Q, Lee JA, Black DL (2007). Neuronal regulation of alternative pre-mRNA splicing. Nat Rev Neurosci.

[20] Nilsen TW, Graveley BR (2010). Expansion of the eukaryotic proteome by alternative splicing. Nature.

[21] Grabowski P (2011). Alternative splicing takes shape during neuronal development. Curr Opin Genet Dev.

[22] Kalsotra A, Cooper TA (2011). Functional consequences of developmentally regulated alternative splicing. Nat Rev Genet.

[23] Savory EA, Zou C, Adhikari BN, Hamilton JP, Buell CR, Shiu SH, Day B (2012). Alternative splicing of a multi-drug transporter from *Pseudoperonospora cubensis* generates an RXLR effector protein that elicits a rapid cell death. PLoS One.

[24] Rodriguez-Kessler M, Baeza-Montanez L, Garcia-Pedrajas MD, Tapia-Moreno A, Gold S, Jimenez-Bremont JF, Ruiz-Herrera J (2012). Isolation of UmRrm75, a gene involved in dimorphism and virulence of *Ustilago maydis*. Microbiol Res.

[25] Ruiz-Roldan C, Pareja-Jaime Y, Gonzalez-Reyes JA, Roncero MI (2015). The transcription factor Con7-1 is a master regulator of morphogenesis and virulence in *Fusarium oxysporum*. Mol Plant Microbe Interact.

[26] Cheon SA, Jung KW, Chen YL, Heitman J, Bahn YS, Kang HA (2011). Unique evolution of the UPR pathway with a novel bZIP transcription factor, Hxl1, for controlling pathogenicity of *Cryptococcus neoformans*. PLoS Pathog.

[27] Bhat RG, Subbarao KV (1999). Host range specificity in *Verticillium dahliae*. Phytopathology.

[28] Klosterman SJ, Subbarao KV, Kang S, Veronese P, Gold SE, Thomma BP, Chen Z, Henrissat B, Lee YH, Park J (2011). Comparative genomics yields insights into niche adaptation of plant vascular wilt pathogens. PLoS Pathog.

[29] Mignone F, Gissi C, Liuni S, Pesole G (2002). Untranslated regions of mRNAs. Genome Biol.

[30] Liu W, Zhou Y, Hu Z, Sun T, Denise A, Fu XD, Zhang Y (2010). Regulation of splicing enhancer activities by RNA secondary structures. FEBS Lett.

[31] Huang C, Xie MH, Liu W, Yang B, Yang F, Huang J, Wu Q, Fu XD, Zhang Y (2011). A structured RNA in hepatitis B virus post-transcriptional regulatory element represses alternative splicing in a sequence-independent and position-dependent manner. FEBS J.

[32] Lejeune F, Maquat LE (2005). Mechanistic links between nonsense-mediated mRNA decay and pre-mRNA splicing in mammalian cells. Curr Opin Cell Biol.

[33] Mendell JT, Sharifi NA, Meyers JL, Martinez-Murillo F, Dietz HC (2004). Nonsense surveillance regulates expression of diverse classes of mammalian transcripts and mutes genomic noise. Nat Genet.

[34] Hillman RT, Green RE, Brenner SE (2004). An unappreciated role for RNA surveillance. Genome Biol.

[35] Kalyna M, Simpson CG, Syed NH, Lewandowska D, Marquez Y, Kusenda B, Marshall J, Fuller J, Cardle L, McNicol J (2012). Alternative splicing and nonsense-mediated decay modulate expression of important regulatory genes in *Arabidopsis*. Nucleic Acids Res.

[36] Ebbole DJ, Jin Y, Thon M, Pan H, Bhattarai E, Thomas T, Dean R (2004). Gene discovery and gene expression in the rice blast fungus, *Magnaporthe grisea*: analysis of expressed sequence tags. Mol Plant Microbe Interact.

[37] Xiong DWY, Ma J, Klosterman SJ, Xiao S, Tian C (2014). Deep mRNA sequencing reveals stage-specific transcriptome alterations during microsclerotia development in the smoke tree vascular wilt pathogen, *Verticillium dahliae*. BMC Genomics.

[38] Wang BB, Brendel V (2006). Genomewide comparative analysis of alternative splicing in plants. Proc Natl Acad Sci U S A.

[39] Jin L-R, Wan P, Kong L-J, Yu D-Z, Huang W, Huang M-S, Wang L (2011). The study on the pathogenicity of differentiation of *Verticullium dahliae* in Hubei province. Cotton Sci.

[40] Trapnell C, Pachter L, Salzberg SL (2009). TopHat: discovering splice junctions with RNA-Seq. Bioinformatics.

[41] Conesa A, Gotz S, Garcia-Gomez JM, Terol J, Talon M, Robles M (2005). Blast2GO: a universal tool for annotation, visualization and analysis in functional genomics research. Bioinformatics.

