# Supplementary material for: Transcriptome analysis reveals the complexity of alternative splicing regulation in the fungus Verticillium dahliae
Source: BMC Genomics. 2017 Feb 6;18:130. doi: 10.1186/s12864-017-3507-y (PMC5294800; doi:10.1186/s12864-017-3507-y)
Supplement: Additional file 1: Figure S1. — Expression profile of the annotated protein-coding genes in the V. dahliae genome revealed by the 1st dataset. Figure S2. The V. dahliae non-coding regions could be enriched in secondary structures. Figure S3. Statistics of the TopHat junction reads. Figure S4. Overlap of the all AS genes. Figure S5. Mutually exclusive exons. Figure S6. Combinatory alternative splicing patterns for V. dahliae pre-mRNA alternative splicing. Figure S7. Representative of biological functions controlled by alternative splicing in the plant pathogen. Figure S8. Representative of biological functions controlled by alternative splicing in the plant pathogen. (DOCX 1433 kb) [file 12864_2017_3507_MOESM1_ESM.docx]

**Transcriptome analysis reveals the complexity of alternative splicing regulation in the fungus *Verticillium dahliae***

Jin et al.,


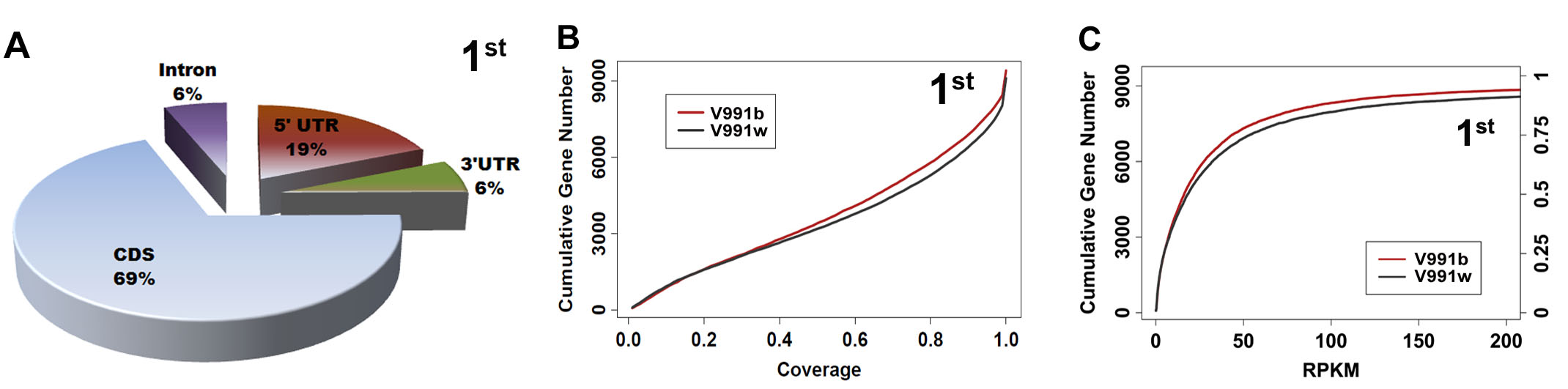


**Figure S1.** Expression profile of the annotated protein-coding genes in the *V. dahliae* genome revealed by the 1^st^ dataset. **(A)** Distribution of the mapped reads in different regions of the expressed genes. The reads of the 1^st^ dataset from both the high- and low-virulence strains were combined for the analysis. **(B)** RNA-seq reads coverage of all expressed genes (1^st^ dataset). Base coverage of each mRNA by the mapped cDNA reads was first calculated, and the number of genes was then plotted against the coverage from 0 (no coverage) to 1 (100% coverage). **(C)** The expression level of all expressed genes represented by RPKM (1^st^ dataset). RPKM stands for mapped reads per kilo base of mRNA per million reads.

**
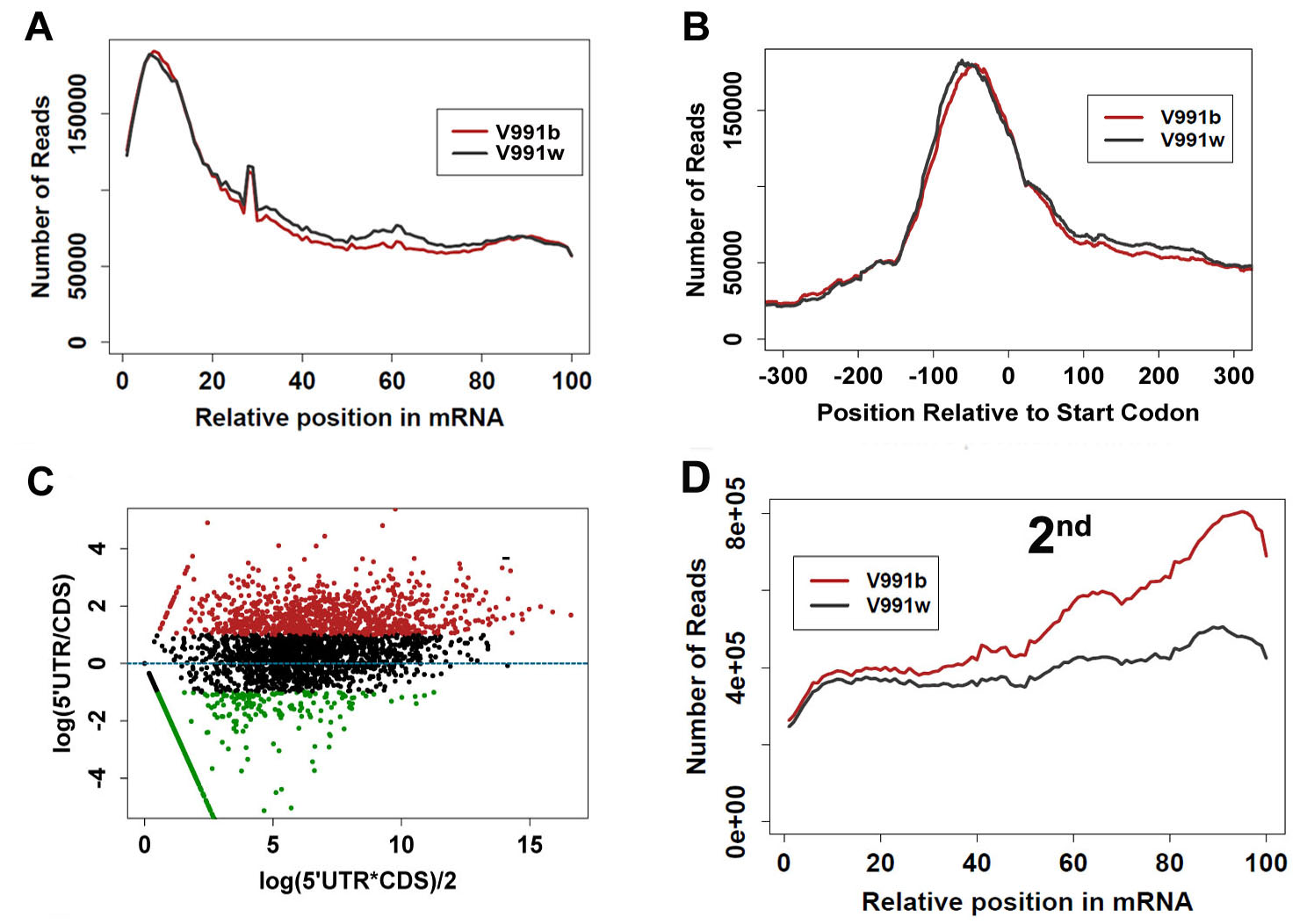
**

**Figure S2.** The *V. dahliae* non-coding regions could be enriched in secondary structures. **(A and D)** Distribution of the mapped reads across the mRNAs from the 5' to 3' end: A-1^st^ dataset; D-2^nd^ dataset. Each mRNA was divided into 100 bins and cDNA reads in each bin was calculated, following by plotting the average read number in each bin along with the mRNA position. **(B)** Plot of the sequence reads of both strains located within 300bp from the translation start codons (1^st^ dataset). **(C)** MA plot of the ratio of cDNA reads in the 5’UTR and CDS regions in all expressed genes containing 5' UTR regions (1^st^ dataset). Y-axis indicates the log_2_ ratio, while X-axis indicates the log_2_ value of the average read concentration in these two regions. Read and green dots show the genes with over-expressed 5' UTR and CDS, respectively.


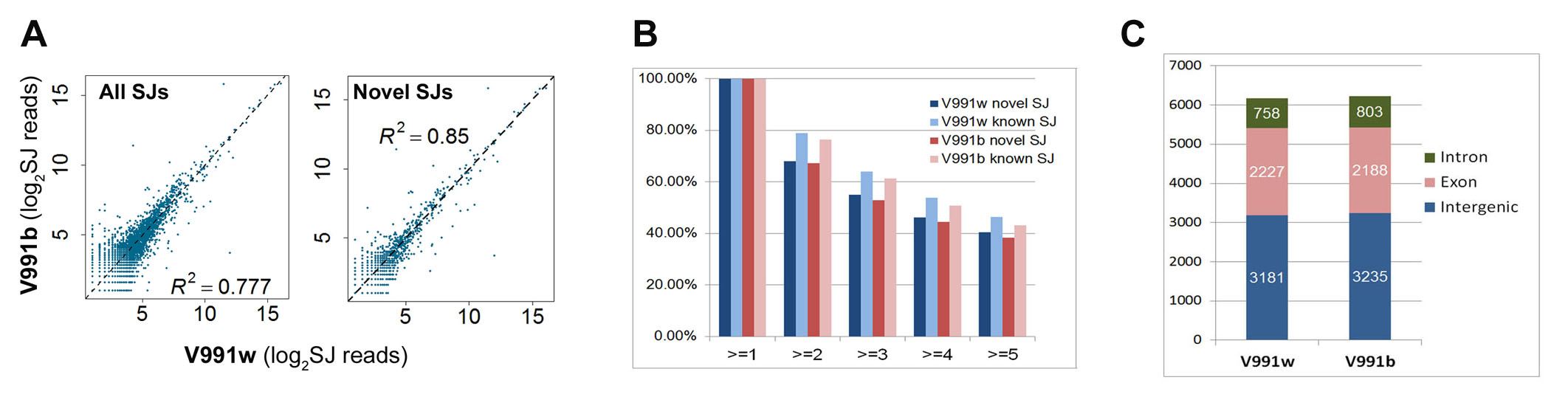


**Figure S3**. Statistics of the TopHat junction reads. (A) Expression correlation of all detected SJ reads and novel SJ reads between two strains; only SJs containing at least one supporting read in both strains were counted, and those existed in one strain but not the other were removed. Supporting reads for all and only novel SJ reads were separately plotted. (B) The percentage of the SJs supported by more than one, two, three and four reads in relative to the total SJs was calculated. The calculation was performed for both the known and novel SJs, and for both *V. dahliae* strains. (C) The genome distribution of the TopHat novel junctions from the 1^st^ dataset was plotted and shown.

**
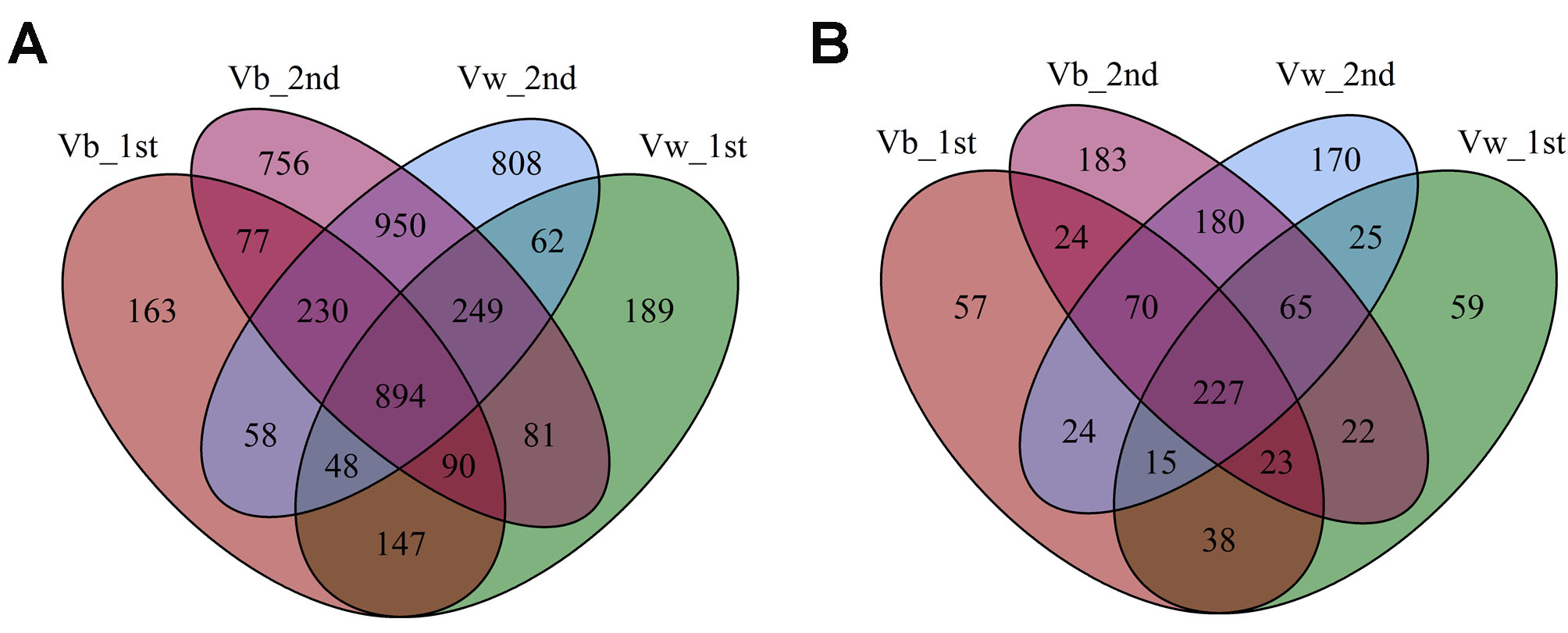
**

**Figure S4**. Overlap of the all AS genes. (**A**) and aJAS genes (**B**) among four transcriptomes of *V. dahliae* strains V991b (Vb) and V991w (Vw).

**
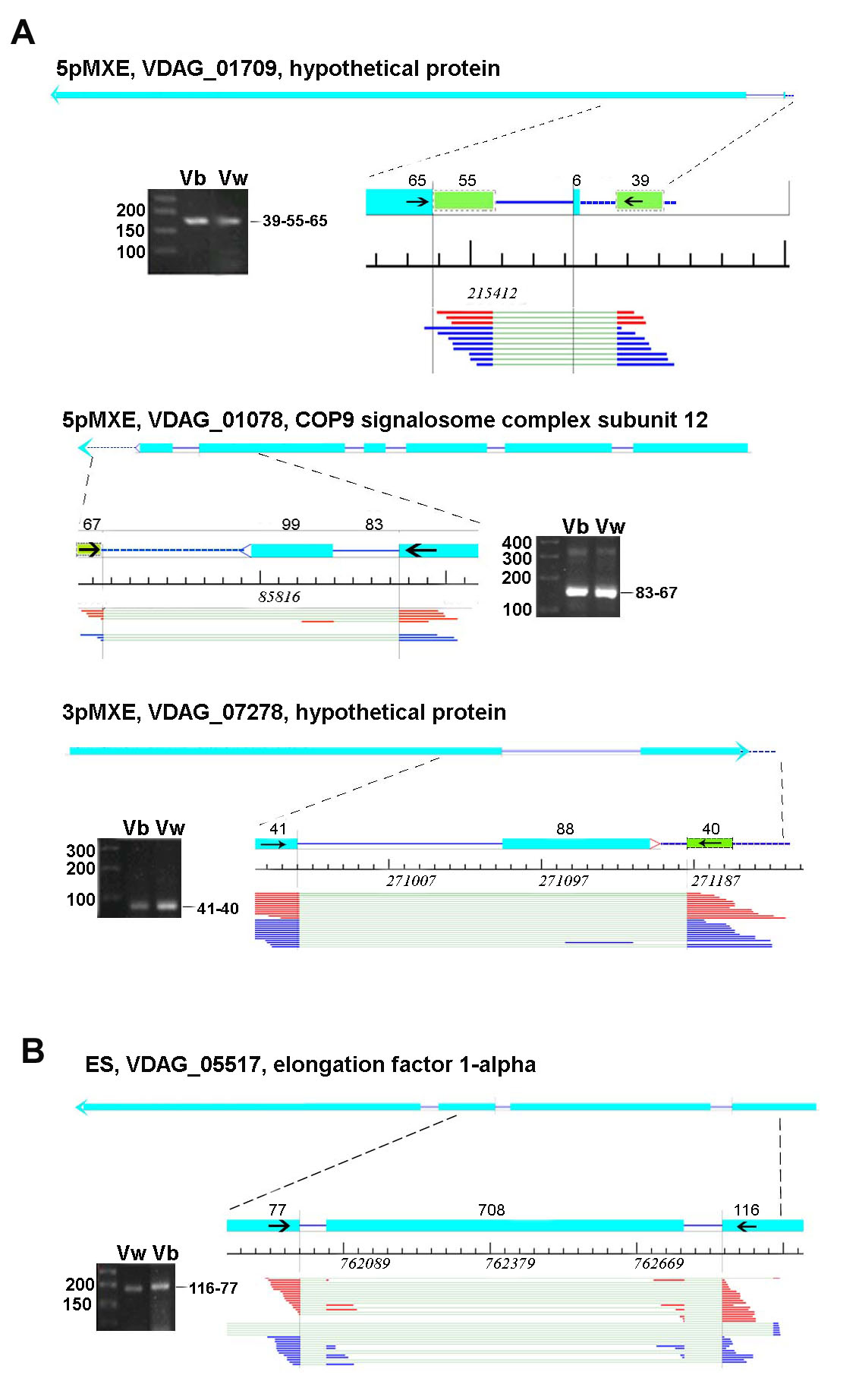
**

**Figure S5**. Mutually exclusive exons. (A) and exon skipping (B) detected for *V. dahliae* pre-mRNA alternative splicing. The figure is labeled as described in Figure 2.

**
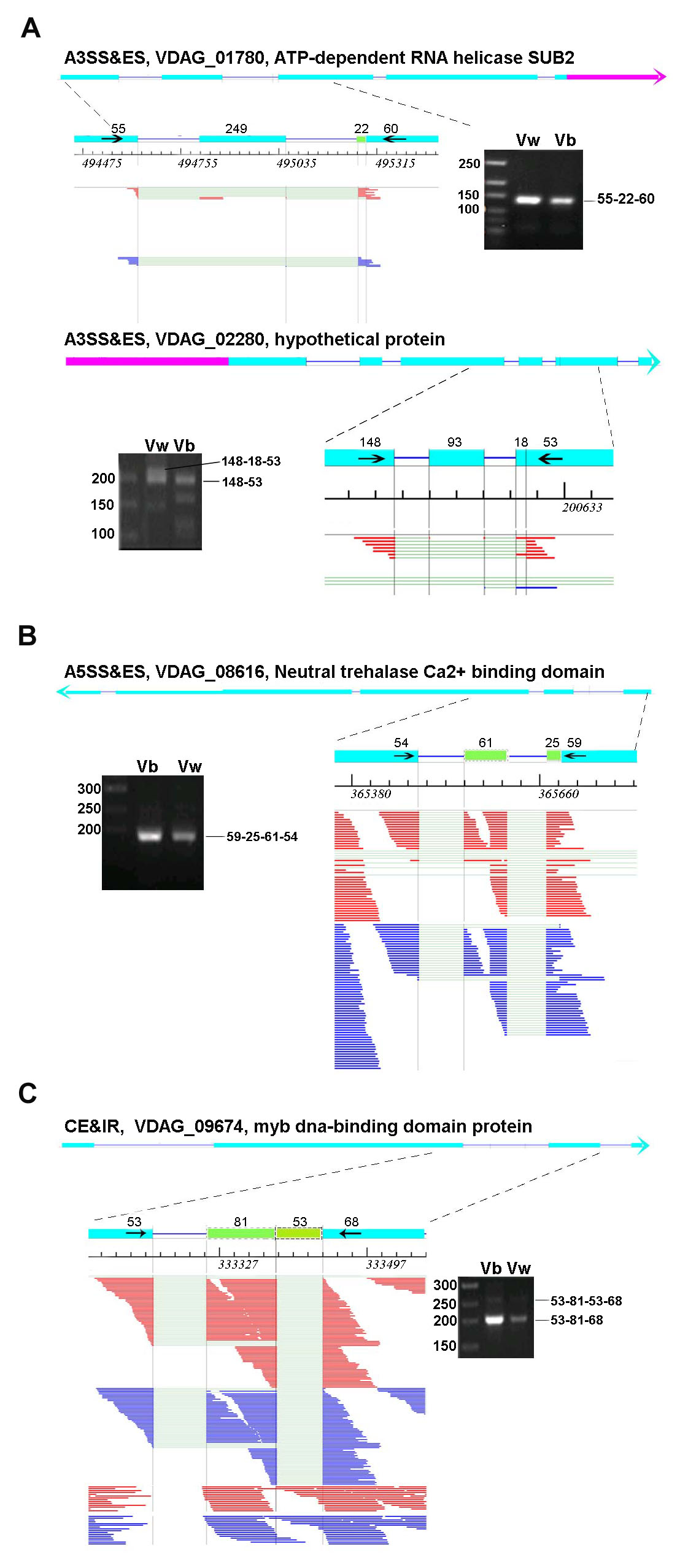
**

**Figure S6**. Combinatory alternative splicing patterns for *V. dahliae* pre-mRNA alternative splicing. (A) A3SS&ES; (B) A5SS&Cassette Exon; (C) Cassette Exon & Intron retention. The figure is labeled as described in Figure 2.


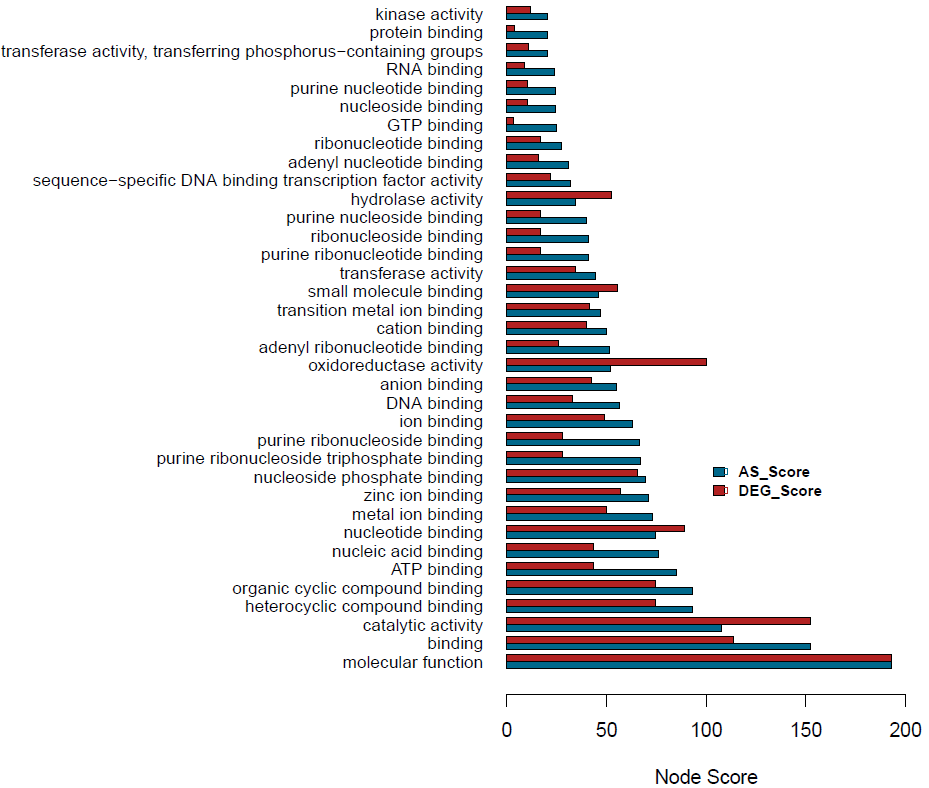


**Figure S7**. Representative of biological functions controlled by alternative splicing in the plant pathogen. All the molecular function terms with a node score larger than 20 were selected for presentation; the node scores of corresponding terms were plotted.


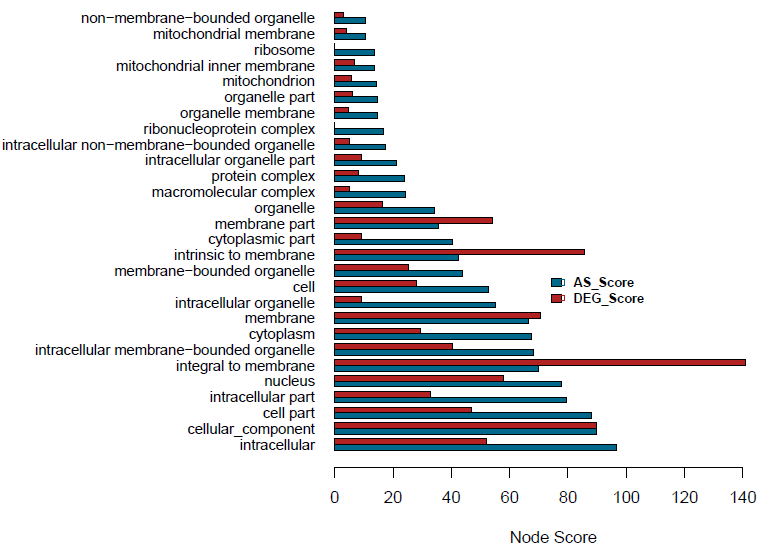


**Figure S8**. Representative of biological functions controlled by alternative splicing in the plant pathogen. All the cellular component terms with a node score larger than 10 were selected for presentation; the node scores of corresponding terms were plotted.
