# Supplementary material for: Transcriptome analysis reveals the complexity of alternative splicing regulation in the fungus Verticillium dahliae
Source: BMC Genomics. 2017 Feb 6;18:130. doi: 10.1186/s12864-017-3507-y (PMC5294800; doi:10.1186/s12864-017-3507-y)
Supplement: Additional file 2: Table S1. — The genic distribution of two sets of transcriptome sequencing reads from the two V. dahliae strains. Table S2. Statistics of the expressed exons, introns and splice junctions (SJ) in the vegetative grown V. dahliae (1st dataset). Table S3. PCR Primers used in this study. (DOCX 32 kb) [file 12864_2017_3507_MOESM2_ESM.docx]

Contents of supplement tables

[Table S1. The genic distribution of two sets of transcriptome sequencing reads from the two *V. dahliae* strains. 2](#_Toc462214428)

[Table S2. Statistics of the expressed exons, introns and splice junctions (SJ) in the vegetative grown *V. dahliae* (1^st^ dataset). 3](#_Toc462214429)

[Table S3. PCR Primers used in this study 3](#_Toc462214430)

## Table S1. The genic distribution of two sets of transcriptome sequencing reads from the two *V. dahliae* strains.

| **A．RNase III fragmentation of PolyA-mRNA and non-directional cDNA library construction, 1^st^ dataset** | | | | | |
| --- | --- | --- | --- | --- | --- |
| Sample | | 5’UTR | 3’UTR | CDS | Introns |
| V991w | | 209,536 | 101,156 | 1,034,829 | 83,518 |
|  |  | (14.66%) | (7.08%) | (72.41%) | (5.85%) |
| V991b | | 207,725 | 118,187 | 955,100 | 88,386 |
|  |  | (15.17%) | (8.63%) | (69.75%) | (6.45%) |
| Total reads | | 417261 | 219343 | 1989929 | 171904 |
| Bases in each region | | 298,696 | 426,826 | 14,984,083 | 1,960,651 |
|  |  | (1.69%) | (2.41%) | (84.80%) | (11.10%) |
| Average depth/base |  | 111.76 | 41.12 | 10.64 | 7.04 |

| **B. Metal ion fragmentation of polyA-mRNA and directional cDNA library construction, 2^nd^ dataset** | | | | |
| --- | --- | --- | --- | --- |
| Sample | 5’UTR | 3’UTR | CDS | Introns |
| V991w | 400,383 | 483,651 | 6,381,550 | 298,717 |
|  | (5.29%) | (6.40%) | (84.36%) | (3.95%) |
| V991b | 384,190 | 734,615 | 7,685,604 | 365,068 |
|  | (4.19%) | (8.01%) | (83.82%) | (3.98%) |
| Total reads | 784,573 | 1,218,266 | 14,067,154 | 663,785 |
| Bases in each region | 298,696  (1.69%) | 426,826  (2.41%) | 14,984,083  (84.80%) | 1,960,651 (11.10%) |
| Average depth/base | 210.13 | 228.34 | 78.10 | 27.08 |

## Table S2. Statistics of the expressed exons, introns and splice junctions (SJ) in the vegetative grown *V. dahliae* (1^st^ dataset).

| **Type** | **All annotated** | **V991b** | | **V991w** | | **Total detected** | |
| --- | --- | --- | --- | --- | --- | --- | --- |
| Supporting reads | | ≥ 1 | ≥ 5 | ≥ 1 | ≥ 5 | ≥ 1 | ≥ 5 |
| Exons | 29,685 | 22,041 | 13,862 | 21,083 | 13,607 | 23,570 (79.40%) | 15,083 (50.81%) |
| Intron | 19,150 | 8,426 | 1238 | 7,931 | 1,116 | 10,511 (54.89%) | 1,472 (7.69%) |
| Predicted SJ | 19,150 | 7,540 | 3243 | 7,420 | 3,438 | 8,804 (45.97%) | 4,049 (21.14%) |
| Novel SJ | **---** | **3,113** | **1192** | **3,083** | **1,244** | **4,790** | **1,750** |
| All SJ | --- | 10,653 | 4435 | 10,503 | 4,682 | 13,594 | 5,799 |

## Table S3. PCR Primers used in this study

| **Primers for alternative splicing validation** | | | |
| --- | --- | --- | --- |
| Name | Sequence | Name | Sequence |
| VDAG_01079F | CGACAATACCGCTAAAGTG | VDAG_01520F | CAGCCATTGCGAAGAAGAT |
| VDAG_01079R | ATGAAGTACCAGTAACGGGA | VDAG_01520R | GTTTCAATGGGGATTTCCAG |
| VDAG_01237F | GGGGATTGAAGAGGATTGAT | VDAG_01780F | CTGAAGCCCGAACTTCTTC |
| VDAG_01237R | TTTTCGGTCTGCTCAACAAA | VDAG_01780R | TCTTTCCGAGACCAGACTT |
| VDAG_02454F | CATTCAACTTCTCTCCCTCG | VDAG_03004F | GACAATCACAAGGAGGAGAC |
| VDAG_02454R | CGTAGACAATGACACCCTG | VDAG_03004R | TTCAACTCGATGGCCTCTAT |
| VDAG_07469F | TTTCAACTTTGGGACCTCTC | VDAG_04099F | CAACAAGCGTGTCACAAAC |
| VDAG_07469R | CAATATCCTCCATCTTGCCA | VDAG_04099R | TAAAAGTTCTGCAGCGCGTA |
| VDAG_08889F | GCATACTCTGGCTCGTTC | VDAG_04392F | GTTGATGCCGTTGTCTACT |
| VDAG_08889R | CTTGGATGAGAGCAGAACC | VDAG_04392R | ATCCCACTTGATTTCCGAAG |
| VDAG_00443F | GATTGTTACCCGGAACAGG | VDAG_04652F | GACCCAGGAGAAGGTCTAC |
| VDAG_00443R | TAGTCAGGAGAAGGAGATGC | VDAG_04652R | GCCAGATTCGTTTTCCATCT |
| VDAG_00578F | TTCGCAAGATTGTCAAGGC | VDAG_05964F | CGCAAGTACGAAATGAACAG |
| VDAG_00578R | ACAAGCCGGGTCCAAAAG | VDAG_05964R | AGTCGATGAGCTTGATAATGT |
| VDAG_08635F | TTGCCAAGGTCAAGGGATTGTTCGG | VDAG_06451F | GTCTGACATGGGCGATGA |
| VDAG_08635R | AGCAGCCGGCGCATCAGG | VDAG_06451R | CTTCTTCTCGTCTTCTTCGG |
| VDAG_07739F | CGTCTCGTGTCGTACATTCTCGGG | VDAG_08324F | GTGCTTCGCTTAATCTGTGA |
| VDAG_07739R | CGTACAAGAAGCCACCAAAGGCA | VDAG_08324R | TCGGCATATTGCCCGAAT |
| VDAG_06386F | CATCGGCATGATGGGCCTCCG | VDAG_08379F | CGCGAAGAAGGATGACAAGA |
| VDAG_06386R | AGTAGTTGCTGAAGCCGCCG | VDAG_08379R | CTGTTCTCCTTCTCAGACTC |
| VDAG_01773F | ACTGCCGCTGTTTGCTCCAGA | VDAG_08400F | CCTGTGCTTCCTCTCCTC |
| VDAG_01773R | GGAGGAACGCTGCCTTGCCA | VDAG_08400R | CATGTTGTCTTTGGCAGATG |
| VDAG_01751F | GAGTCTCGCGACCCCCGTGA | VDAG_10099F | AAACTCATGAATGGCTTTGG |
| VDAG_01751R | TGGCAACGGCCTCCTCATCTG | VDAG_10099R | GAGCGAATTGAACGGTTAGA |
| VDAG_01706F | CGGAAGTACTCGGAGTCGTAGGCG | VDAG_01690F | GCCGGGCCAGGCAATGACTC |
| VDAG_01706R | CGCAAACGCCGTGTGTCCCT | VDAG_01690R | CAACCCGTGCGGACCTTGGC |
| VDAG_01696F | GCGACACAGGACGCGGAGG | VDAG_00171F | CCCAGCAGGCGGCAGACTTG |
| VDAG_01690R | AGGAATACCACGGATTCTTCCTCACC | VDAG_00171R | GCAGACCTCATCGCTTCCGGG |
| VDAG_05625F | CCAGATCGCGAGGTCGGGC | VDAG_05964F | CCGCGACACCACGGGTGAG |
| VDAG_05625R | GCCTTGGTCTTCTCGTATTCTTCGGT | VDAG_05964R | TGGCGCATGATCTGGACTTCCT |
| VDAG_06555F | GCCCCATTCCCACGTGTGGTC | VDAG_01137F | AGCCTCGGAAACAGTACCAGACC |
| VDAG_06555R | ACGGGACCATCCATGCCTTCG | VDAG_01137R | GCGGAGGGGGCCATTGTAGC |
| VDAG_01312F | TCGACCAGCACGACAGTCACC | VDAG_01709F | TCGGGGAGAGCTCGTGGGAT |
| VDAG_01312R | GGTCGTCACCACTGCGCTCG | VDAG_01709R | TCAACCCCAACCGAGCAAGGC |
| VDAG_01078F | CGGGGAATGCGCCGTTCTTG | VDAG_05517F | AATACACGACGCCCTGTGGTACT |
| VDAG_01078R | GTTTGCCGCGGCCATCAGTC | VDAG_05517R | GGCCCTCCGTTGGACTTGGG |
| VDAG_01780F | AAGCCCGAACTTCTTCGTGCCA | VDAG_02280F | CCTTCACGGGCGAGCCCAAC |
| VDAG_01780R | CGGCCGTCTTTCCGAGACCAG | VDAG_02280R | GCGTTCTGCCCTCTTCTCCCC |
| VDAG_08616F | TCGACGATGCCAAGACGTACTACA | VDAG_09674F | CAGCGCCTCAAGCAGATGCGA |
| VDAG_08616R | GGTCGACTGGACATTGACGAGGA | VDAG_09674R | ACCTTCTGCCCAATAACCTTCCACTT |
